# Identification of Cyclic Dipeptides and a New Compound (6-(5-Hydroxy-6-methylheptyl)-5,6-dihydro-2*H*-pyran-2-one) Produced by *Streptomyces fungicidicus* against *Alternaria solani*

**DOI:** 10.3390/molecules27175649

**Published:** 2022-09-01

**Authors:** He Liu, Mengnan An, Hongyang Si, Yuhang Shan, Chuantao Xu, Gang Hu, Yunbo Xie, Dongyang Liu, Shujun Li, Rui Qiu, Chong Zhang, Yuanhua Wu

**Affiliations:** 1 College of Plant Protection, Shenyang Agricultural University, Shenyang 110866, China; 2 Luzhou Branch of Sichuan Province Tobacco Company, Luzhou 646000, China; 3 Sichuan Province Tobacco Company, Chengdu 610017, China; 4 Liangshanzhou Branch of Sichuan Province Tobacco Company, Xichang 615000, China; 5 Key Laboratory for Green Preservation & Control of Tobacco Diseases and Pests in Huanghuai Growing Area, Tobacco Research Institute, Henan Academy of Agricultural Sciences, Xuchang 461000, China

**Keywords:** *Streptomyces fungicidicus*, anti-*A. solani* activity, isolation and purification, structural determination, novel compound

## Abstract

As an important microbial resource, *Actinomycetes*, especially *Streptomyces,* have important application values in medicine and biotechnology. *Streptomyces fungicidicus* SYH3 was isolated from soil samples in tomato-growing areas and showed good inhibitory effects on *Alternaria solani* in tomato. To obtain pure active compounds, SYH3 fermentation broth was subjected to XAD-16 macroporous resin and silica gel column chromatography. Combined with the repeated preparation and separation of preparative high-performance liquid chromatography (HPLC), a total of four monomer compounds were obtained after activity tracking. Compound **4** was identified as a new six-membered lactone ring compound named 6-(5-hydroxy-6-methylheptyl)-5,6-dihydro-2*H*-pyran-2-one by 1D and 2D nuclear magnetic resonance (NMR) data and mass spectrometry (MS). The other three active compounds belong to the cyclodipeptide, and their half maximal inhibitory concentration (IC_50_) values against *A. solani* were 43.4, 42.9, and 30.6 μg/mL, respectively. Compound **4** significantly inhibited the spore germination and induced swollen and deformed local hyphae of *A. solani* with an IC_50_ value of 24.9 μg/mL. Compound **4** also had broad-spectrum antifungal activity and had a good antifungal effect on the tested plant-pathogenic fungi. The modes of action of new compound (**4**) still require further investigation, representing a novel and effective anti-fungal agent for future application.

## 1. Introduction

*Actinomycetes* are one of the most widely used biocontrol microorganisms; have great practical value; and are closely associated with human health, agriculture, and biotechnology [1]. Approximately 8,000 bioactive substances have been discovered in microorganisms, of which approximately 70% are derived from *Actinomycetes* [2]. New biological pesticides developed by using the secondary metabolites of *Actinomycetes* have become an important component of pollution-free pesticides and provide the direction for the future development of green pesticides, which have the advantages of low residue, low pollution, less drug resistance, low cost, and easy production [3,4]. *Streptomyces* is a genus with the most species in the *Actinomycetes*, involving approximately 600 kinds, and accounting for approximately 90% of *Actinomycetes*. Approximately 2/3 of the known antibiotics are derived from *Actinomyces*, and approximately half of all antibiotics are derived from *Streptomyces* [5].

For instance, mitomycin is a metabolite isolated from the fermentation broth of *Streptoverticillium rimofaciens* Niida that inhibits the synthesis of fungal proteins and has a good control effect on powdery mildew diseases [6]. *Streptomyces kasugaensis* can produce kasugamycin, which inhibits the protein synthesis of many microorganisms and is currently used as a biopesticide to control rice blast and other diseases caused by *Pseudomonas* on crops [7]. Polyoxins B and D isolated from the metabolites of *Streptomyces cacaoi* var. *asoensis* were reported to interfere with the formation of the fungal cell wall of *Rhizoctonia solani* by inhibiting its chitinase activity [8].

The main chemical structures of the active metabolites secreted by *Streptomyces* include polyketides, macrolides, cyclic dipeptides, pyrones, and indoles [9]. For instance, phenyltetracenoid polyketides isolated from *Streptomyces morookaense* exhibited inhibitory activity against *Staphylococcus aureus* and *Enterococcus faecalis* [10]. *Streptomyces fungicidicus* can metabolize to produce the tetraene antifungal antibiotic, fungicidin, which has the activity of inhibiting filamentous fungi, yeast, and protozoa. *Streptomyces misionensis* V16R3Y1 metabolizes cyclic dipeptide (L-Leu-L-Pro) with broad-spectrum resistance to phytopathogenic fungi [11]. Antimycin is a quinone antibiotic produced by the metabolism of *Streptomyces* and can inhibit the synthesis of pathogen RNA and protein due to their unique chemical structure [12].

Tomato early blight caused by *Alternaria solani* is one of the important diseases in tomato cultivation, which can lead to detaching of the leaves, drying of branches, and premature fruit drop, and the resulting fruit yield losses often amount to 50–86% [13,14]. The main chemical agents that have been applied to control tomato early blight include trifloxystrobin, tebuconazole [15], oxathiapiprolin, and benthiavalicarb [16], while the currently used biocontrol agents include *Bacillus* [17] and *Trichoderma* [18], which are still very limited. In this study, *A. solani* was used as an indicator fungus, and four active compounds were obtained from a *Streptomyces fungicidicus* SYH3 fermentation broth by a series of separation and purification procedures. The structures of the four compounds were established by 1D and 2D NMR data, MS, and by comparison with the literature data. One of these active compounds (**4**) was determined to be a new compound 6-(5-hydroxy-6-methylheptyl)-5,6-dihydro-2*H*-pyran-2-one, and we applied for a Chinese patent (number: CN111320597A). The other three compounds (**1**–**3**) were previously reported as cyclic dipeptide compounds, and their IC_50_ values against *A. solani* were 43.4, 42.9, and 30.6 μg/mL, respectively. The new compound **4** has a significant inhibitory effect on *A. solani* and can significantly affect the spore germination and mycelial morphology of the pathogen.

## 2. Results

### 2.1. Anti-A. solani Activity of the SYH3 Fermentation Broth and Its Purified Products

**Figure 1:**
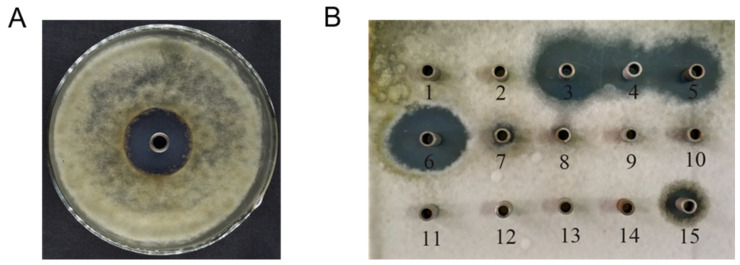
Anti-fungal activity of *S. fungicidicus* SYH3 fermentation broth against *A. solani* (**A**) and its active components obtained by isolation and purification (**B**).

Bioassay-guided fractionation allows for the evaluation of the activity of the components at each stage of the separation test and the tracing of the most active component to achieve a purposeful separation of the active product [19]. After the mycelium was removed from the fermentation broth of *S. fungicidicus* SYH3 by centrifugation and filtration, the diameter of inhibition zone that suppress growth of *A. solani* was 39 mm (Figure 1A). After resin adsorption, extraction, and silica gel column chromatography of the fermentation liquid, 15 components were obtained, among which component 6 had the most significant anti-fungal effect against *A. solani* with an inhibition zone diameter of 35.2 mm. Therefore, this component was then selected for further separation and purification to obtain active compounds (Figure 1B). Component 6 was repeatedly collected and separated by preparative HPLC, and four monomer compounds, **1**, **2**, **3**, and **4**, were obtained. The results showed that the IC_50_ value of compound **4** was 24.7 μg/mL, and the IC_50_ values of the other three compounds were 43.4 μg/mL, 42.9 μg/mL, and 30.6 μg/mL, respectively. Meanwhile, the inhibitory rate of compound **4** on *A. solani* reached 93.8% at the concentration of 128 μg/mL, which was similar to the inhibitory rate after rapamycin treatment at the same concentration (Table 1).

### 2.2. Structural Identification of the Isolated Four Active Monomeric Compounds

**Figure 2:**
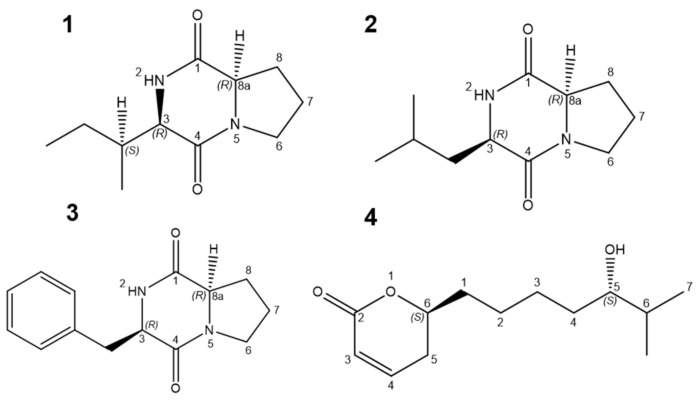
Schematic diagram of the molecular structure of the four monomer compounds. Compounds **1**–**4**.

Determining the specific active components of the biocontrol microorganism metabolites is critical for the research, development, and practical application of biological pesticides. In this study, the structures of the four active compounds (**1**-**4**) were studied by analyzing the mass spectra of the compounds, combined with the hydrogen spectrum, the carbon spectrum, the distortionless enhancement by polarization transfer (DEPT) spectrum, and the two-dimensional spectrum in the nuclear magnetic resonance spectrum. The compound **1** was determined to be a cyclic (L-proline-L-isoleucine) dipeptide (Cyclo-(L-Pro-L-Ile)) based on the spectral data, the public SCI Finder database (SciFinder. Available online: https://scifinder-n.cas.org/ (accessed on 16th March 2022)), and compared with the literature data. Its molecular formula was C_11_H_18_N_2_O_2_, and it was named (3*R*,8a*R*)-3-((*S*)-*sec*-butyl)hexahydropyrrolo [1,2-*a*]pyrazine-1,4-dione (Figure 2, Figures S1–S6). Meanwhile, the compound **2** was determined to be a cyclic (L-proline-L-leucine) dipeptide (Cyclo-(L-Pro-L-Leu)). Its molecular formula was C_11_H_18_N_2_O_2_, and it was named (3*R*,8a*R*)-3-isobutylhexahydropyrrolo [1,2-*a*]pyrazine-1,4-dione (Figure 2, Figures S6–S11); the compound **3** was determined to be a cyclic (L-phenylalanine-L-proline) dipeptide (Cyclo-(L-Phe-L-Pro)). Its molecular formula was C_14_H_16_N_2_O_2_, and it was named (3*R*,8a*R*)-3-benzylhexahydropyrrolo [1,2-*a*]pyrazine-1,4-dione (Figure 2, Figures S12–S17).

**Figure 3:**
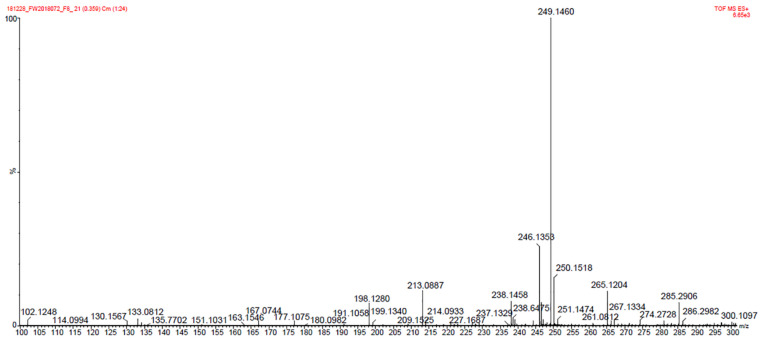
HRESIMS spectra of compound **4**.

According to HRESIMS m/z = 249.1460 [M + Na]^+^, (Figure 3) [M + Na]^+^ (calculated: C_13_H_22_O_3_Na, 249.1467), the molecular formula of compound **4** was determined to be C_13_H_22_O_3_, its melting point was 114–116.5 °C, and its optical rotation was [α]^20^_D_ = −31.5 (*c* 2.8, CHCl_3_). The results of the ^1^H NMR and ^13^C NMR spectra show that the compound has two tertiary methyl groups [δH 0.87 (t, J = 6.2 Hz, 3H), δH 1.13 (d, J =6.4 Hz, 3H)]; furthermore, there is one ester bond carbonyl carbon signal [δC 173.2 (C = O)] and a set of double bond carbon signals [δC 156.3 and δC 121.6] in the compound. The ^13^C NMR showed that it has 13 C signals, based on DEPT and HSQC. The results showed that the compound contains two primary carbons (δC = 19.5, 14.6), five secondary carbons (δC = 33.2, 25.0, 29.6, 27.0, 32.4), five tertiary carbons (δC = 121.6, 156.3, 83.4, 71.7, 40.0), and one quaternary carbon (δC = 173.2). Further analysis using HMBC showed that δH 0.87 (C-13) is related to δC 71.7 (C-10), δC 40.0 (C-11); δH 1.13 (C-13) is related to δC 71.7 (C-10), δC 40.0 (C-11); δH 3.65 (C-10) is related to δC 32.4 (C-9), δC 40.0 (C-11), δC 14.4 (C-13), δC 19.5 (C-12); δH 1.46 (C-6) is related to δC 29.6 (C-7); δH 5.04 (C-5) is related to δC 33.2 (C-4); δC 156.3 (C-3) is related to δC 25.0 (C-6); δH 1.76 (C-4) is related to δC 156.3 (C-3); δC 83.4 (C-5) is related to δC 25.0 (C-6); δH 7.45 (C-3) is related to δC 83.4 (C-5), δC 121.6 (C-2), and δC 173.2 (C-1) (Figures S19–S23). According to these data, the new compound **4** was chemically named 6-(5-hydroxy-6-methylheptyl)-5,6-dihydro-2*H*-pyran-2-one (Figure 2). We applied for a Chinese national patent for compound **4** and were officially authorized (number: CN111320597A).

### 2.3. Effects of Compound 4 on Spore Germination and Germ Tube Morphology

**Figure 4:**
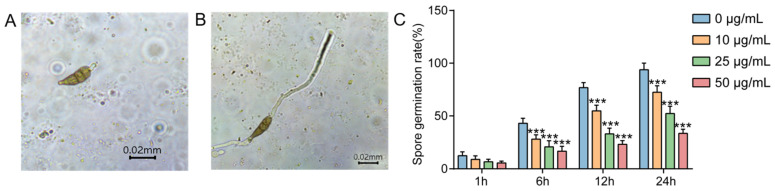
Effects of compound **4** on spore germination of *A. solani.* (**A**) The fungal spores treated with compound **4** at the IC_50_ concentration for 12 h. (**B**) The untreated spore germination (control). (**C**) Spore germination rate when treated with different concentrations of compound **4** at different hours. *** indicates a significant difference (*p* < 0.001).

**Figure 5:**
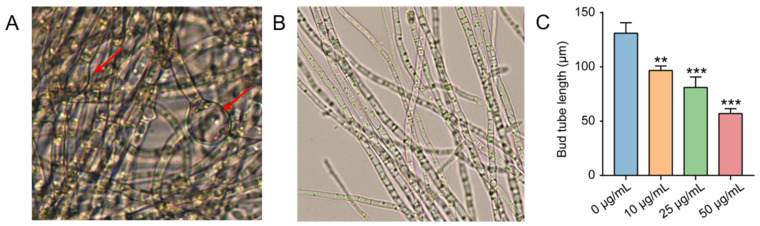
Effects of compound **4** on germ tube elongation and morphology of *A. solani.* (**A**) Mycelial morphology after compound **4** treatment (24 h). (**B**) Morphology of normal hyphae. (**C**) Length of germ tube treated with different concentrations of compound **4** (24 h). ** indicates a significant difference (*p* < 0.01) and *** indicates a significant difference (*p* < 0.001).

The effect of pesticides on the spore germination of the pathogens is the basis for inhibiting the occurrence and development of diseases, while the effects of pesticides on the mycelium of pathogens can affect the invasion and pathogenesis on host plants [20,21]. In this study, the results showed that the germination of *A. solani* was significantly inhibited when the pathogen was treated with the new compound **4** at the IC_50_ concentration for 12 h (Figure 4A,B), and the germination rate was 53.5% at 24 h, which was significantly lower than that of the control group (96.3%) (Figure 4C). The results indicated that the compound **4** treatment suppressed the spore germination rate of *A. solani*, in a concentration-dependent manner (Figure 4C). Compared with the control group, the hyphae in the compound **4** treatment group became thicker and denser, and the local swelling deformed (Figure 5A,B). Under the compound **4** treatment at the IC_50_ concentration, the mycelium length was reduced by approximately 50 µm compared with that in the control (0 μg/mL) group (Figure 5C).

### 2.4. Antimicrobial Spectrum of Compound 4 against Test Pathogens

To determine the practical value of compound **4** as a broad-spectrum antimicrobial, we evaluated its inhibitory activity against several other plant fungal pathogens. The results showed that compound **4** had a good inhibitory effect on most of the tested strains, and the diameters of the inhibition zones were larger than 20 mm. Specifically, the inhibitory effect of the compound on *Rhizoctonia solani* was the most significant, and the diameter of the inhibition zone was 43.2 ± 0.9 mm, while *Gibberella zeae* and *Phytophora parasitica* were slightly less sensitive to compound **4**, with inhibition zone diameters of 21.3 ± 0.6 and 20.6 ± 0.8 mm, respectively (Table 2).

## 3. Discussion

*S. fungicidicus* was reported to confer good control effects on a variety of horticultural plant fungal diseases, and its fermentation metabolites show broad-spectrum antimicrobial activity [22,23]. A previous study showed that *S. fungicidicus* YH9407 isolated from soil samples can produce tetraene antibiotics [24]. *S. fungicidicus* isolated from the sedimentary soil of the Pacific Ocean floor produces a low-toxicity, antifouling metabolite [25]. Additionally, *S. fungicidicus* MML1614, which produces a highly active proteolytic enzyme, was screened from the bottom sedimentary soil of the Bay of Bengal in the Indian Ocean [23]. Enramycin, a polypeptide antibiotic produced by the metabolism of *S. fungicidicus*, has strong inhibitory effects on Gram-positive bacteria [26]. In this study, three cyclic dipeptide compounds were isolated from *S. fungicidicus* SYH3: Cyclo-(L-Phe-L-Pro), Cyclo-(L-Pro-L-Leu), Cyclo-(L-Pro-L-Ile), and a new compound: 6-( 5-hydroxy-6-methylheptyl)-5,6-dihydro-2*H*-pyran-2-one.

Compounds **1**-**3** are cyclic dipeptides formed by the condensation of two α-amino acids through peptide bonds. According to their characteristic structures, it was determined that the three compounds belong to the diketopiperazines (DKPs). Notably, DKPs have become an important pharmacophore in medicinal chemistry because of their stable six-membered ring skeleton structure, which has attracted increasing attention [27]. In recent years, a large number of active natural products with the structure of DKPs have been isolated from the bacteria, *Actinomycetes,* and fungi from marine sources [28,29,30]. The DKPs exhibit a variety of biological and pharmacological activities, and can be synthesized into anti-cancer, anti-tumor [31,32], anti-inflammatory analgesics, stimulants, and anti-hypertensive drugs in medicine [33]. In addition, some of the DKPs are signaling molecules of intercellular communication, which can regulate the LuxR-mediated bacterial quorum-sensing system and control the formation of biofilms by interfering with the information exchange between the microorganisms [34]. Here, the DKPs’ compound**1**-Cyclo-(L-Pro-L-Ile), was reported to be isolated from *Galactomyces geotrichum* [35], *Lactobacillus plantarum* [36], *Pseudomonas fluorescens,* and *Pseudomonas alcaligenes* cell-free culture supernatants [37]. To our knowledge, such cyclic dipeptides have not been reported to be produced from *S. fungicidicus*. The compound **2**-Cyclo-(L-Pro-L-Leu) has good antagonistic activity against *Bacillus subtilis*, *Escherichia coli*, *Staphylococcus aureus*, and *Pseudomonas aeruginosa* [38,39,40,41]. In particular, the effect of the compound on the spore germination and hyphal growth of *Pyricularia oryzae* was comparable to the inhibitory effect on *A. solani* in this study [42]. The compound **3**-Cyclo-(L-Phe-L-Pro) is mainly derived from marine microorganisms, such as marine mollusks [43], marine-derived *Bacillus cereus* [44], and marine sponge *Dysidea* sp. [45], while it has not been shown that the compound can be produced from *S. fungicidicus*. Collectively, the anti-fungal effects of compound **1**-**3** on *A. solani* have not been reported, and their modes of action still require further investigation in future works.

In addition, we isolated and identified a new compound, 6-(5-hydroxy-6-methylheptyl)-5,6-dihydro-2*H*-pyran-2-one, which was effective on the spore germination and mycelial growth of *A. solani*. The six-membered lactone ring structure of this compound is a common core pharmacodynamic skeleton in drugs [46]. The hypolipidemic drug lovastatin and the anti-falciparum malaria drug artemisinin both contain the structural backbone of a six-membered lactone ring [47]. Therefore, we speculate that the six-membered lactone ring structure of the new compound **4** is the main active functional structure. To our knowledge, there are no reports on the resistance of six-membered lactone ring compounds on plant diseases. Therefore, to further clarify the mechanism of action of the compounds, the metabolic process and the action targets of the compounds should be investigated in any future study. In addition, whether the new compound can induce host defense responses is also an interesting topic, and a corresponding investigation should also be performed in future work.

In this study, 15 components were obtained after resin adsorption, extraction, and silica gel column chromatography from the fermentation broth of *S. funcidicus* SYH3. Four compounds, including a new compound **4**, were further isolated and purified from the most effective component 6 of the fermentation broth. Importantly, the new compound had a significant inhibitory effect on the germination of *A. solani* spores and the growth of hyphae, and its chemical structure was confirmed by mass spectrometry and one- and two-dimensional nuclear magnetic resonance. Nevertheless, we did not purify and investigate the potentially effective compounds from other components (e.g., components 3, 4, 5, and 15 also conferred considerable anti-fungal effects) in this study, which remain to be further elucidated in future works. In conclusion, this study provides new insights into the study of the active products of *S. funcidicus*, demonstrating the activity of DKPs and six-membered lactone ring compounds against plant fungal diseases. A feasibility prediction analysis of hexavalent lactones as anti-fungal lead compounds is presented.

## 4. Materials and Methods

### 4.1. Microorganisms, Media, and Culture Conditions

The *S. fungicidicus* strain was collected and isolated from soil samples from tomato-growing areas in Shenyang, Liaoning, China (41°48′ N, 123°25′ E). The pathogenic fungi *A. solani*, *Gibberella zeae*, *Botrytis cinerea*, *Fusarium solani*, *Candida albicans*, *Colletotrichum capsica*, and *Bipolaris maydis* were provided by the College of Plant Protection, Shenyang Agricultural University, China. *Alternaria alternata* and *Rhizoctonia solani* were isolated from the tobacco-growing area in Luzhou, Sichuan, China (28°52′ N, 105°26′ E). *Fusarium oxysporum* and *Phytophora parasitica* var. *nicotiana* were isolated from the tobacco-growing area in Xinyang, Henan, China (31°48′ N, 114°05′ E). The strains above were stored on PDA medium (potato dextrose agar, 20 g agar powder, 20 g D-glucose, 200 g potato) at 4 °C. The strains, activated at 28 °C, were used in subsequent measurements in anti-microbial experiments, and for the preparation of spore suspensions.

To obtain the fermentation culture of *S. fungicidicus*, first, the fermentation seed liquid was prepared according to a previous study [48]. Then, the seed liquid, after culturing for 18 h, was inoculated into a 2 L Erlenmeyer flask containing 500 mL of MS medium (Sigma-Aldrich, Darmstadt, Germany) at an inoculation amount of 1%. The fermentation was shaken at 28 °C, 180 rpm for 5 d. Then, the fermentation culture was centrifuged at 10,000 rpm and 4 °C for 15 min to remove mycelium. The obtained supernatant fermentation broth was used for the subsequent biocontrol, isolation, and purification experiments.

### 4.2. Isolation and Purification of S. fungicidicus Metabolites

First, 5% XAD-16 macroporous adsorption resin was added into the fermentation broth collected by centrifugation above. The active substances were adsorbed by shaking at 28 °C, 150 rpm for 4 h. The resin was eluted with methanol 4 times and dried and concentrated with a rotary evaporator to obtain the crude extract. The extract was preseparated into 15 components by silica gel (75–150 μm) column chromatography using a CH_2_Cl_2_/MeOH gradient. Combined with the results of the bioactivity test, component 6 was further purified. The gradient elution was performed by preparative HPLC using water/methanol as the mobile phase (Packing: Ulimate^®®^ AQ-C18 (Waters, Milford, CT, USA), 10 μm; flow rate: 1 mL/min; detection wavelength: 210 nm). The single substance collected above was dried and used for subsequent activity determination and structure determination.

### 4.3. Determination of Anti-A. solani Activity of Fermentation Broth and Purified Products

The inhibitory activity of the fermentation broth, 15 components (which were obtained by silica gel column chromatography), and 4 compounds (which were obtained from the preparation and separation of component no. 6) were determined using *A. solani* as the indicator pathogen. Referring to the relevant literature [49], the Oxford cup method was used to measure the diameter of the inhibition zone and determine the anti-microbial activity in the samples. A conidial suspension (10^4^ CFU/mL) of *A. solani* was prepared as described [50]. A five hundred microliter *A. solani* spore suspension was mixed in 100 mL PDA medium at about 45 °C and separated into petri dishes. Two hundred microliters of fermentation broth or solution of the tested component were added to an Oxford cup on a petri dish. After culturing at 28 °C for 3 d, the diameter of the inhibition zone was measured with a Vernier caliper. To determine the isolated compound’s IC_50_, the compound was added into an appropriate amount of PDA medium to adjust to the final concentrations of 16, 32, 64, and 128 μg/mL. The mycelial plugs (5 mm diameter) of *A. solani* were placed in the center of a PDA plate and incubated in a 28 °C incubator. The growth of fungal colonies was measured 3 days post-inoculation (dpi) and three biological replicates were performed. Sterile water treatment was used as the mock group and kasugamycin (Shengyuan, Guizhou, China) treatment was used as the control group. The inhibition rate of the compounds on the growth of *A. solani* mycelium was calculated by the following formula. The IC_50_ values of compounds were calculated by GraphPad Prism (7.0), referring to existing studies [51].

Net growth = average colony diameter − cake diameter(1)

Anti-fungal rate (%) = [(control colony net growth − treated colony net growth)/control colony net growth] × 100(2)

### 4.4. Determination of Anti-A. solani Activity of Fermentation Broth and Purified Products

The structure of the compounds was determined using nuclear magnetic resonance (NMR) spectroscopy (BRUKER AVANCE III HD 400MHz; Bruker, Rheinstetten, Germany). The NMR spectrometer using CDCl_3_ was deployed to measure ^1^H and ^13^C and 2D NMR. All of the spectra were recorded at 23 °C. The one-dimensional ^1^H NMR experiments, as well as the two-dimensional 1H-1H correlation spectroscopy, Distortionless enhancement by polarization transfer (DEPT), heteronuclear single-quantum correlation (HSQC), and ^1^H-^13^C heteronuclear multiple-bond correlation (HMBC) experiments were performed according to Bruker standard pulse sequences. Chemical shifts were reported relative to the solvent peaks (CDCl_3_: ^1^H d 7.24 and ^13^C d 77.23). The mass spectra (MS) were determined on a JEOL JMS-SX/SX102A four-sector tandem MS (JEOL, Ltd., Tokyo, Japan), coupled with an electrospray source. The probe voltage was maintained at 2.5 kV, the cone voltage was maintained at 18 V, and the extractor voltage at 2 V. The source temperature was kept at 100 °C, and the desolvation temperature was 300 °C. The compound melting points (m.p.) were measured by an MP30 melting point apparatus (Mettler Toledo, Zurich, Switzerland). The optical rotations were measured using an MCP 4100 smart polarimeter (Anton Paar, Graz, Austria).

### 4.5. Effects of Compound 4 on Spore Germination and Germ Tube Elongation in A. solani

Referring to existing research methods [52], the conidia suspension of *A. solani* was mixed with an appropriate amount of the compound to the final concentration of 10, 25, and 50 μg/mL, respectively. The agent-treated conidia were incubated at 28 °C on a concave glass slide before observation. The spore germination rate was assessed while the length and morphology of the germ tube were observed and conducted at 1 h, 6 h, 12 h, and 24 h post inoculation (hpi) by optical microscope Model Eclipse E200 (Nikon, Japan). All assays were performed at least 3 times, with approximately 150–200 spores observed for each assay. The spore germination rate (%) was calculated as follows: number of spore germinations/total number of spores in field ×100.

### 4.6. Inhibitory Activity of Compound 4 against Different Pathogenic Microorganisms

To investigate whether compound **4** has broad-spectrum resistance to pathogenic fungi, we referred to the Oxford Cup method of method 4.3 and evaluated the anti-fungal effect of compound **4** against 10 common pathogenic fungi, including *Gibberella zeae*, *Botrytis cinerea*, *Fusarium solani*, *Candida albicans*, *Colletotrichum capsica*, *Bipolaris maydis*, *Alternaria alternata*, *Rhizoctonia solani*, *Fusarium oxysporum*, and *Phytophora parasitica* var. *nicotiana*. An Oxford cup was placed in the center of the PDA medium containing the fungal suspension, and 200 μL of compound **4** at IC_50_ concentration was added. The diameter of the clear zone of inhibition was measured and recorded after 3 d of incubation at 28 °C. The above experiments were performed in three biological replicates.

## 5. Patents

We applied for a Chinese national patent for compound **4** (6-(5-hydroxy-6-methylheptyl)-5,6-dihydro-2*H*-pyran-2-one) and were officially authorized (number: CN111320597A).

## Figures and Tables

**Table 1:** Determination of inhibition rate and IC_50_ values of four monomer compounds against *A. solani.* Mean ± standard deviation indicated by different letters are significantly different according to the Duncan’s multiple range test at *p* < 0.05.

Compounds	Mycelial Growth Inhibition Rate (%)					IC_50_ (μg/mL)
	0 μg/mL	16 μg/mL	32 μg/mL	64 μg/mL	128 μg/mL	
Kasugamycin	0	67.3 ± 5.1	85.1 ± 5.0	92.6 ± 2.5	94.7 ± 3.1	14.5 ± 0.9 d
**1**	0	31.8 ± 7.4	46.0 ± 4.5	69.0 ± 4.2	81.4 ± 2.9	43.4 ± 2.9 a
**2**	0	37.0 ± 5.3	48.2 ± 4.1	73.3 ± 4.5	81.8 ± 4.1	42.9 ± 1.2 a
**3**	0	20.8 ± 4.1	45.9 ± 8.2	68.8 ± 5.0	75.1 ± 3.7	30.6 ± 1.9 b
**4**	0	66.1 ± 5.4	79.2 ± 4.5	89.3 ± 8.8	93.8 ± 3.6	24.7 ± 0.8 c

**Table 2:** Antimicrobial spectrum of compound **4** against test pathogens.

Pathogens	Inhibition Zone Diameter (mm)
*Alternaria solani*	38.3 ± 0.7
*Gibberella zeae*	21.3 ± 0.6
*Phytophora parasitica*	20.6 ± 0.8
*Botrytis cinerea*	26.8 ± 1.1
*Fusarium solani*	31.2 ± 1.5
*Alternaria alternata*	24.7 ± 0.9
*Rhizoctonia solani*	43.2 ± 0.9
*Colletotrichum capsici*	25.0 ± 0.5
*Fusarium oxysporum*	36.8 ± 1.6
*Bipolaris maydis*	36.4 ± 1.2
*Candida albicans*	39.5 ± 1.0

## Data Availability

Not applicable.

## Supplementary Materials

The following are available online at https://www.mdpi.com/article/10.3390/molecules27175649/s1, Figure S1: HRESIMS spectra of compound **1**; Figure S2: ^1^H NMR (400 MHz) spectra of compound **1** in CDCl3; Figure S3: ^13^C NMR (100 MHz) spectra of **1** in CDCl_3_; Figure S4: DEPT spectra of compound **1** in CDCl_3_; Figure S5: HSQC spectra of compound **1** in CDCl_3_; Figure S6: HMBC spectra of compound **1** in CDCl_3_; Figure S7: HRESIMS spectra of compound **2**; Figure S8: ^1^H NMR (400 MHz) spectra of compound **2** in CDCl3; Figure S9: ^13^C NMR (100 MHz) spectra of **2** in CDCl_3_; Figure S10: DEPT spectra of compound **2** in CDCl_3_; Figure S11: HSQC spectra of compound **2** in CDCl_3_; Figure S12: HMBC spectra of compound **2** in CDCl_3_; Figure S13: HRESIMS spectra of compound **3**; Figure S14: ^1^H NMR (400 MHz) spectra of compound **3** in CDCl_3_; Figure S15: ^13^C NMR (100 MHz) spectra of **3** in CDCl_3_; Figure S16: DEPT spectra of compound **3** in CDCl_3_; Figure S17: HSQC spectra of compound **3** in CDCl_3_; Figure S18: HMBC spectra of compound **3** in CDCl_3_; Figure S19: ^1^H NMR (400 MHz) spectra of compound **4** in CDCl_3_; Figure S20: ^13^C NMR (100 MHz) spectra of **4** in CDCl_3_; Figure S21: DEPT spectra of compound **4** in CDCl_3_; Figure S22: HSQC spectra of compound **4** in CDCl_3_; Figure S23: HMBC spectra of compound **4** in CDCl_3_.

## Sample Availability

Samples of the compounds are available from the authors.
