# Supplementary figures and images for: Identification of Cyclic Dipeptides and a New Compound (6-(5-Hydroxy-6-methylheptyl)-5,6-dihydro-2H-pyran-2-one) Produced by Streptomyces fungicidicus against Alternaria solani
Source: Molecules. 2022 Sep 1;27(17):5649. doi: 10.3390/molecules27175649 (PMC9458140; doi:10.3390/molecules27175649)

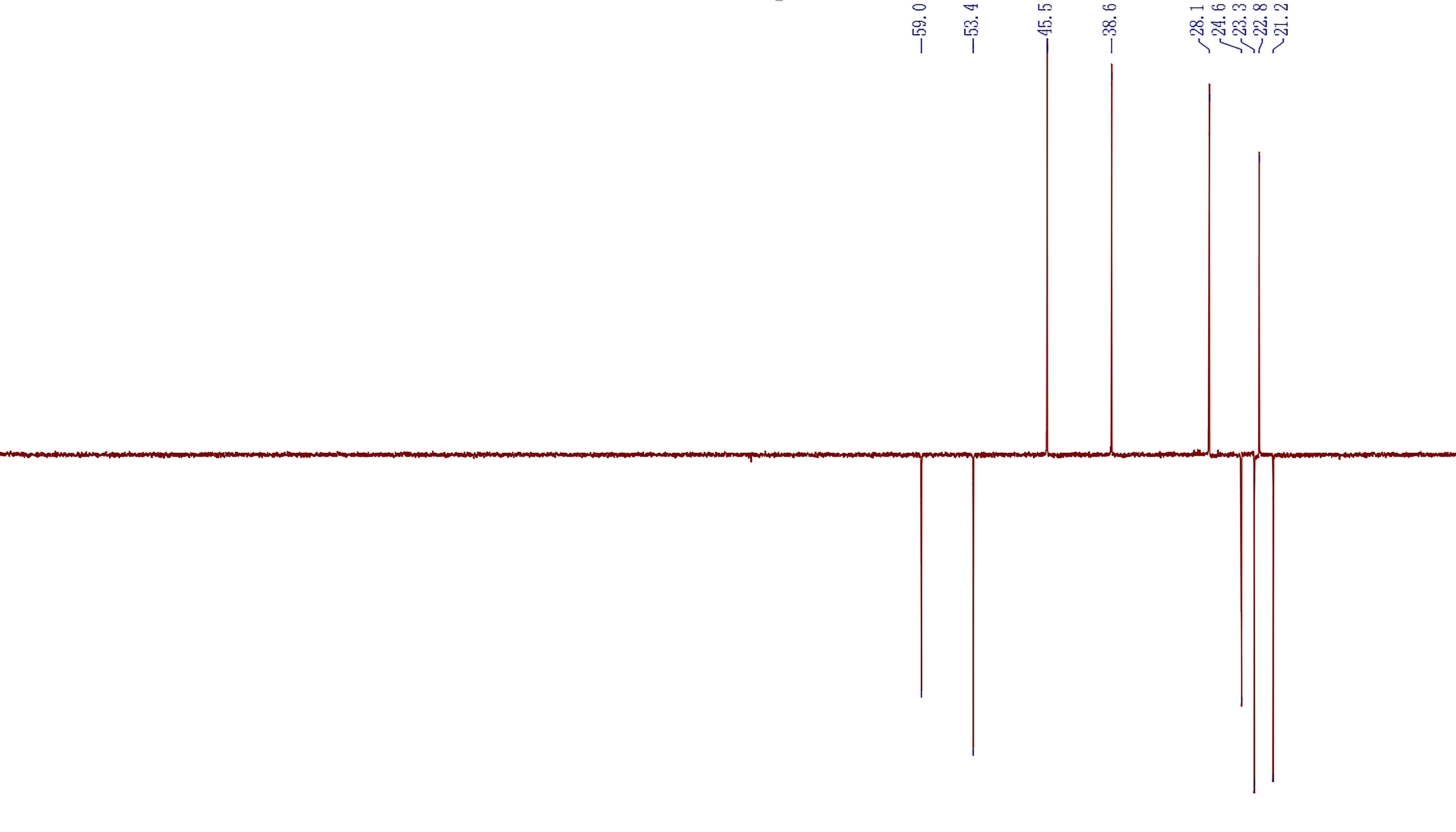

Supplement: Supplementary file 1 [file molecules-27-05649-s001.zip › S-PNG-8-15/Figure.S10 DEPT spectra of compound 2 in CDCl3.png]

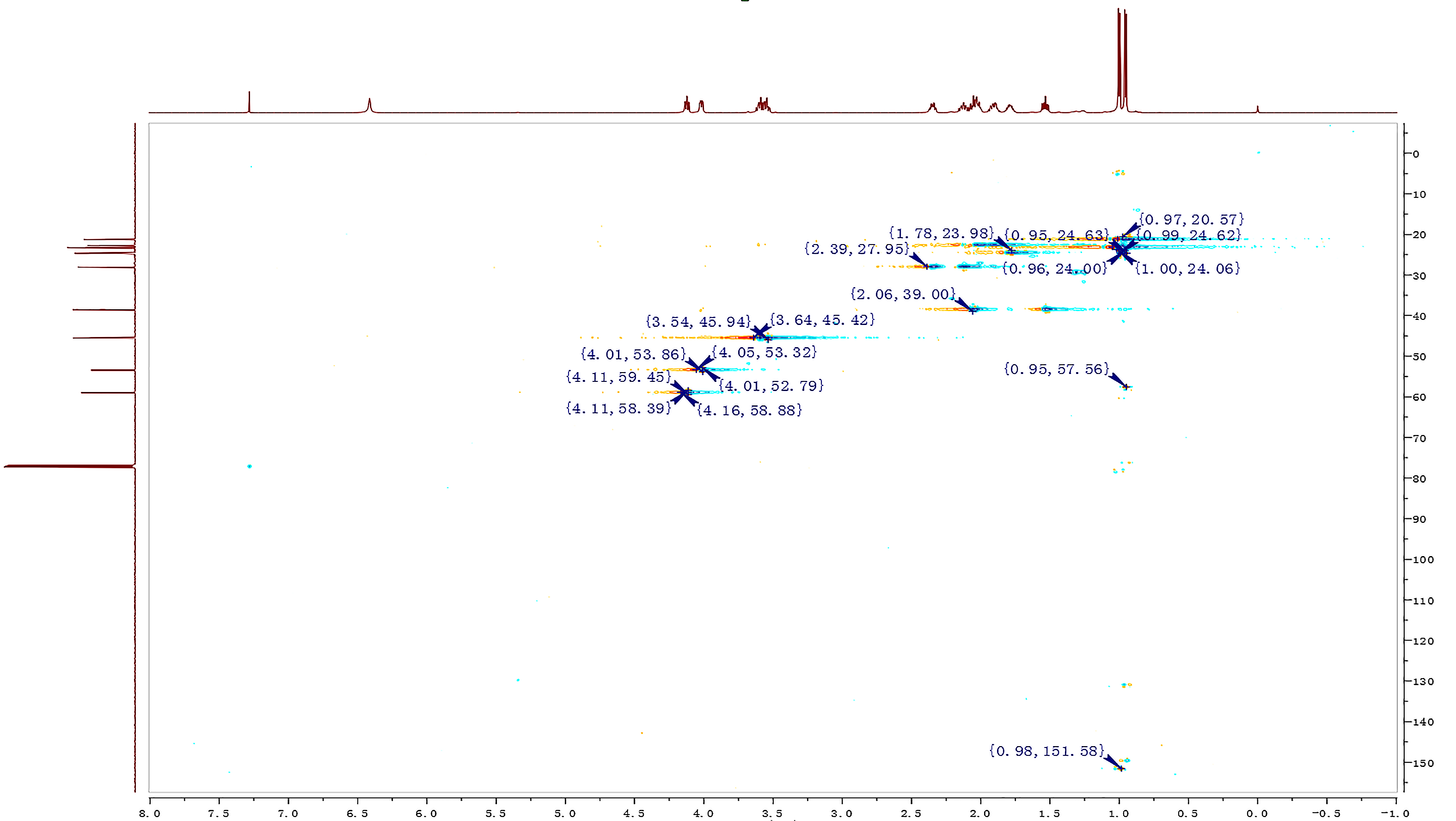

Supplement: Supplementary file 1 [file molecules-27-05649-s001.zip › S-PNG-8-15/Figure.S11 HSQC spectra of compound 2 in CDCl3.png]

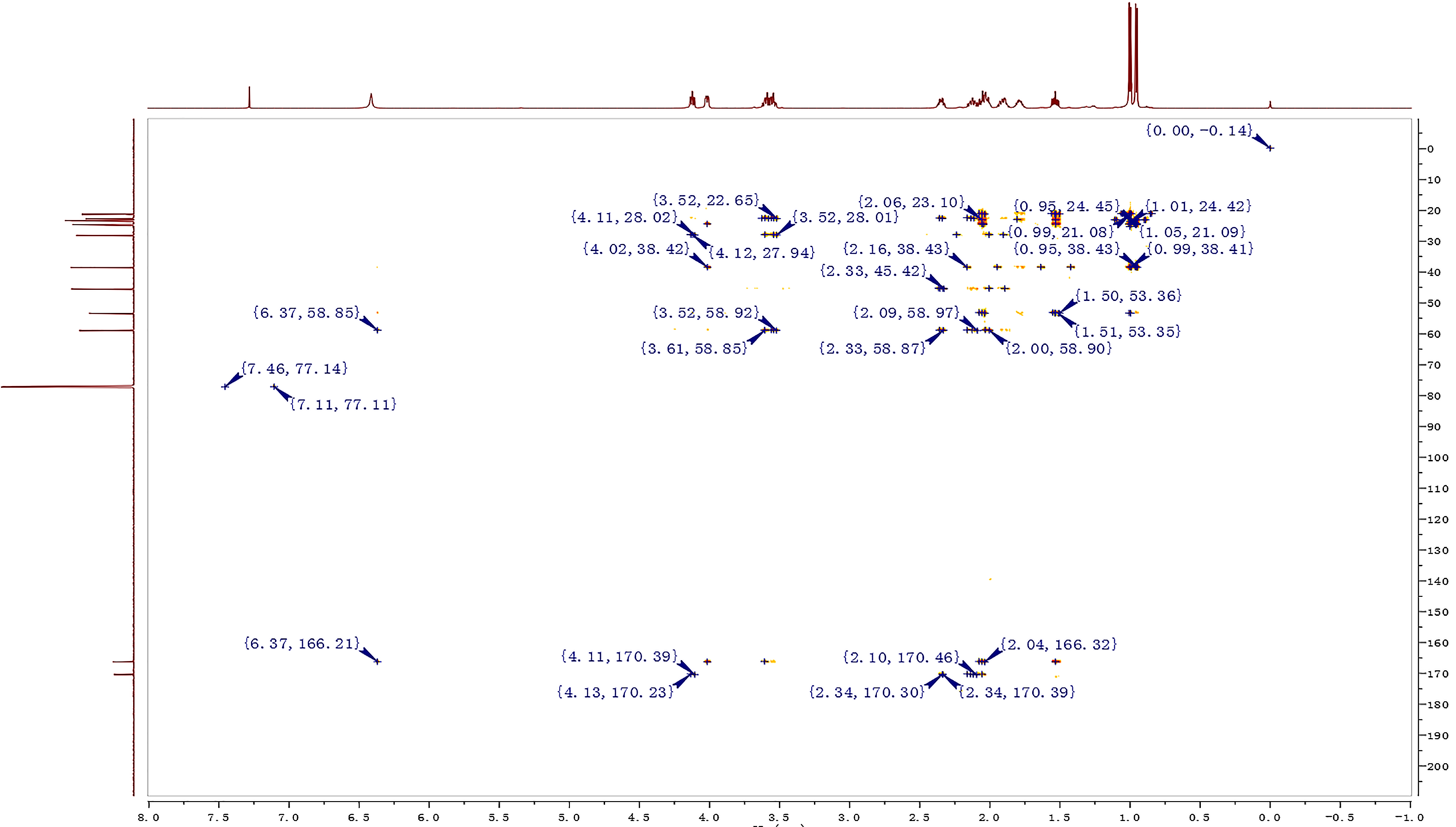

Supplement: Supplementary file 1 [file molecules-27-05649-s001.zip › S-PNG-8-15/Figure.S12 HMBC spectra of compound 2 in CDCl3.png]

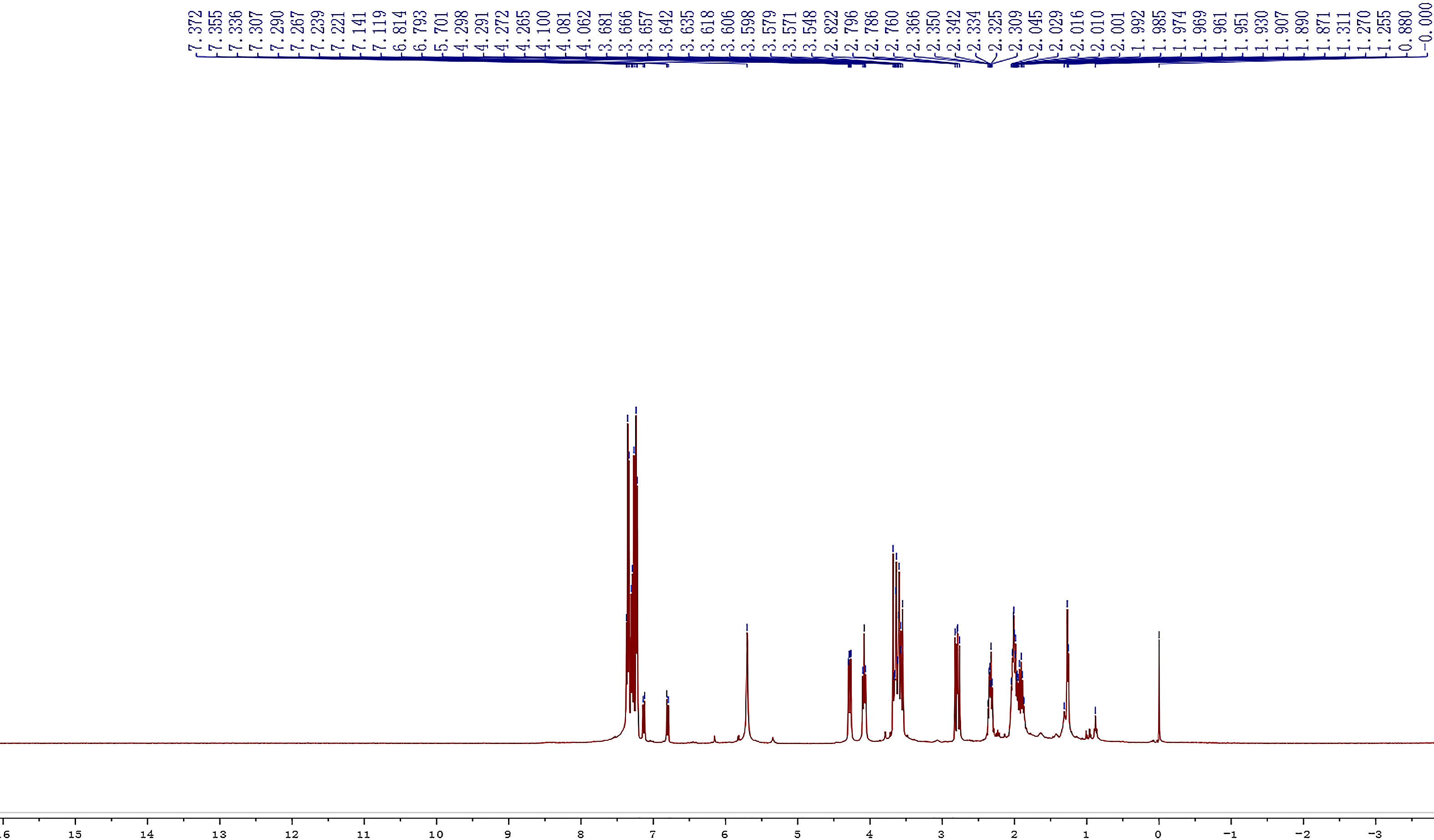

Supplement: Supplementary file 1 [file molecules-27-05649-s001.zip › S-PNG-8-15/Figure.S14 1H NMR (400 MHz) spectra of compound 3 in CDCl3.png]

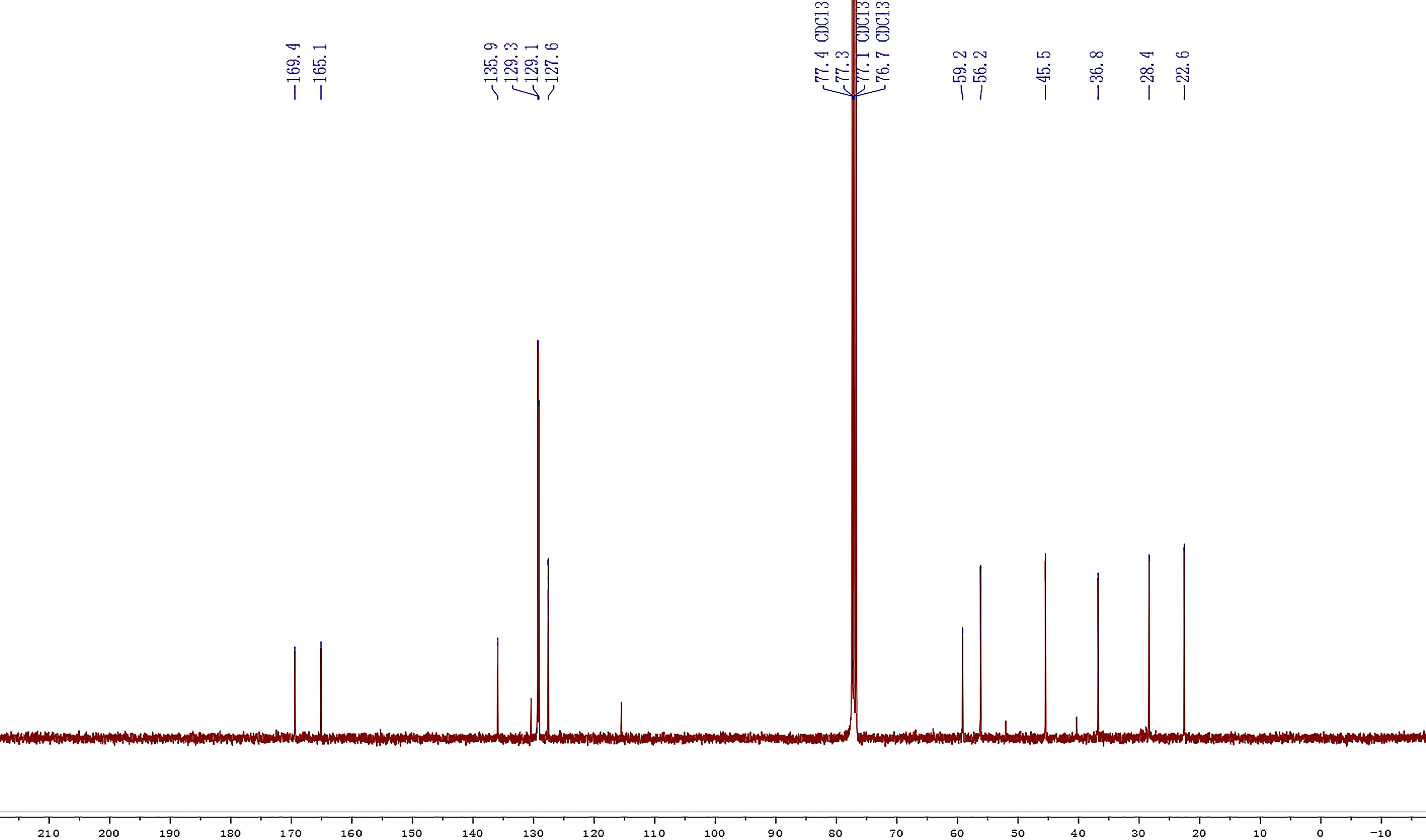

Supplement: Supplementary file 1 [file molecules-27-05649-s001.zip › S-PNG-8-15/Figure.S15 13C NMR (100 MHz) spectra of compound 3 in CDCl3.png]

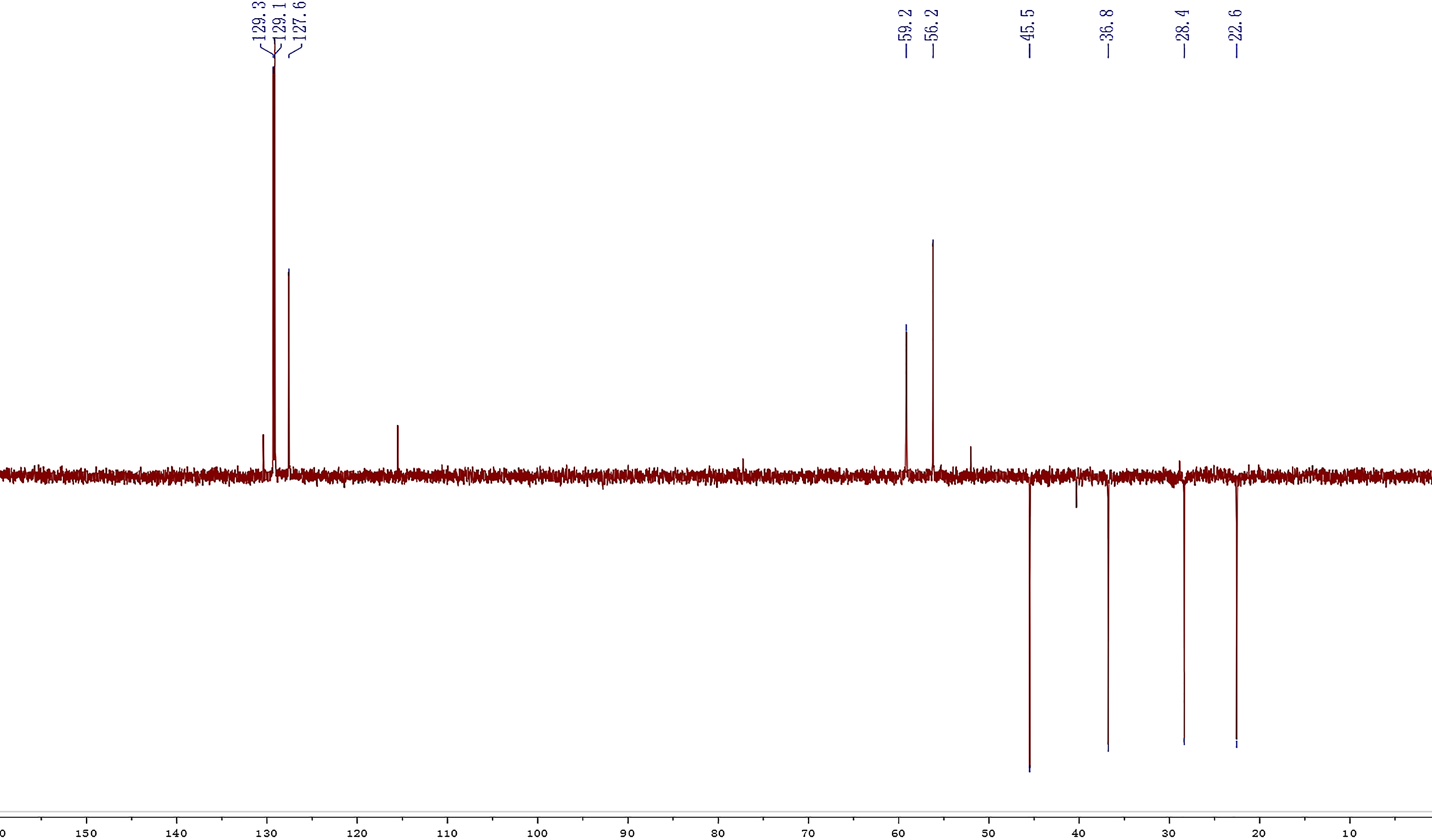

Supplement: Supplementary file 1 [file molecules-27-05649-s001.zip › S-PNG-8-15/Figure.S16 DEPT spectra of compound 3 in CDCl3.png]

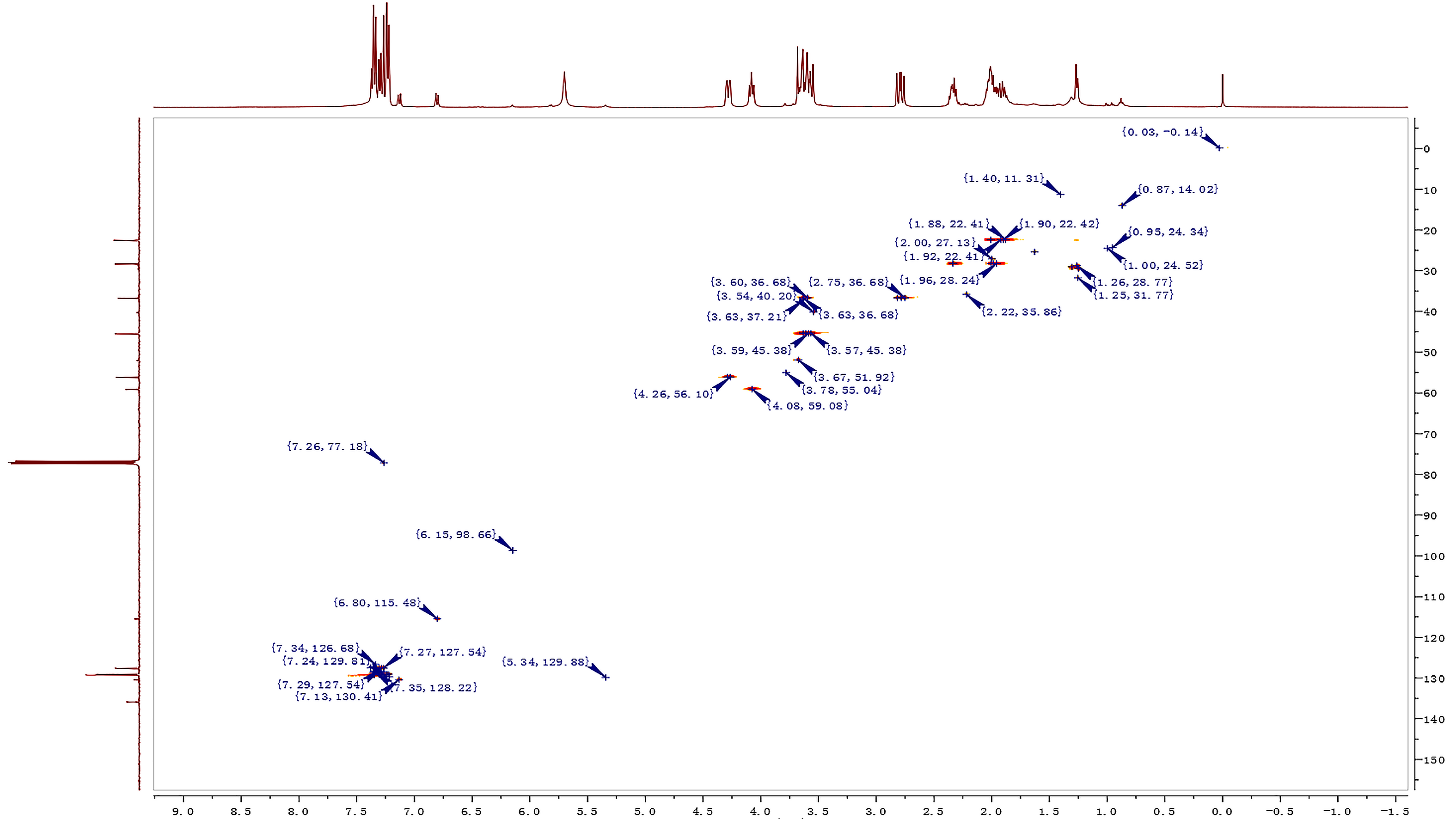

Supplement: Supplementary file 1 [file molecules-27-05649-s001.zip › S-PNG-8-15/Figure.S17 HSQC spectra of compound 3 in CDCl3.png]

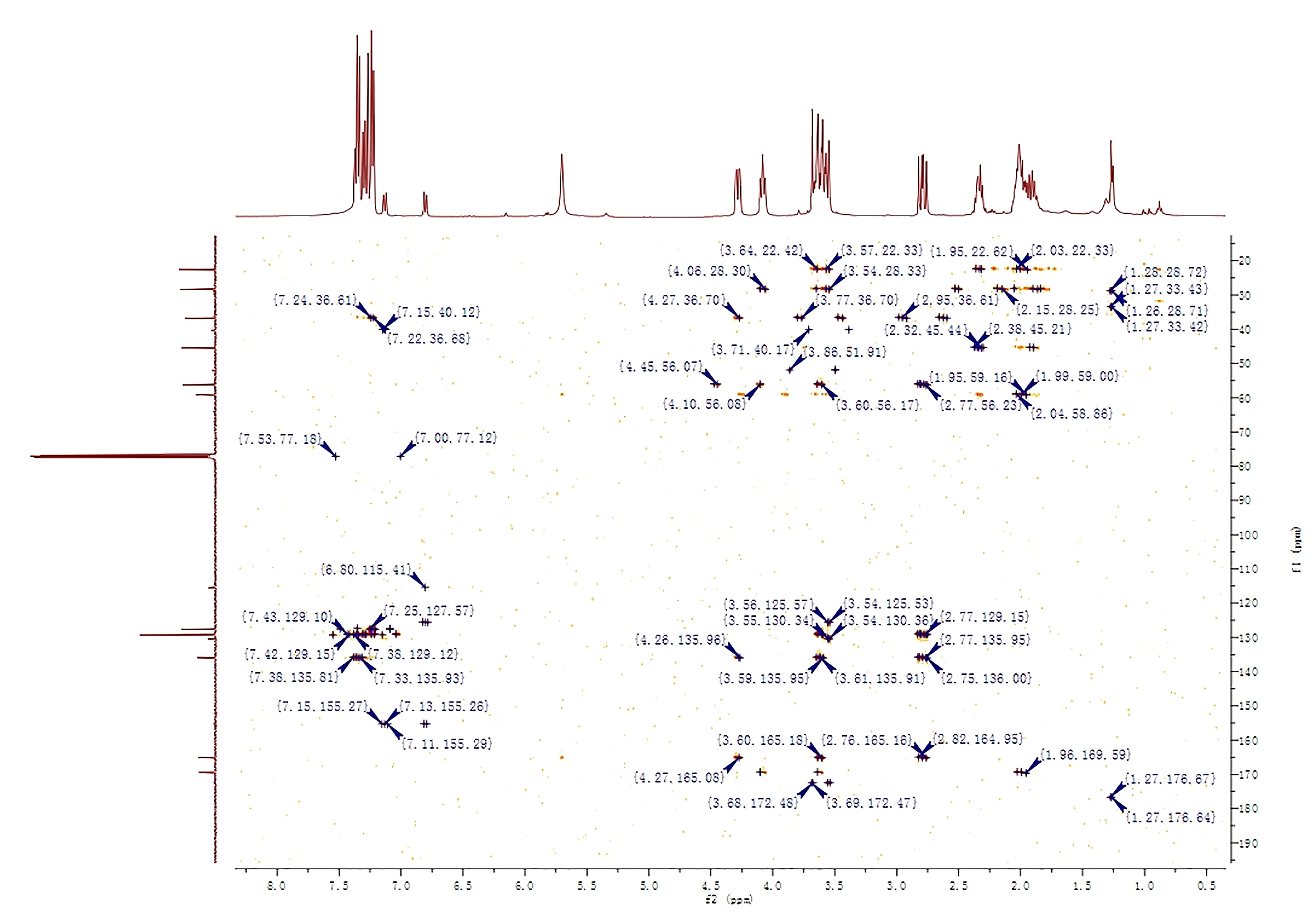

Supplement: Supplementary file 1 [file molecules-27-05649-s001.zip › S-PNG-8-15/Figure.S18 HMBC spectra of compound 3 in CDCl3.png]

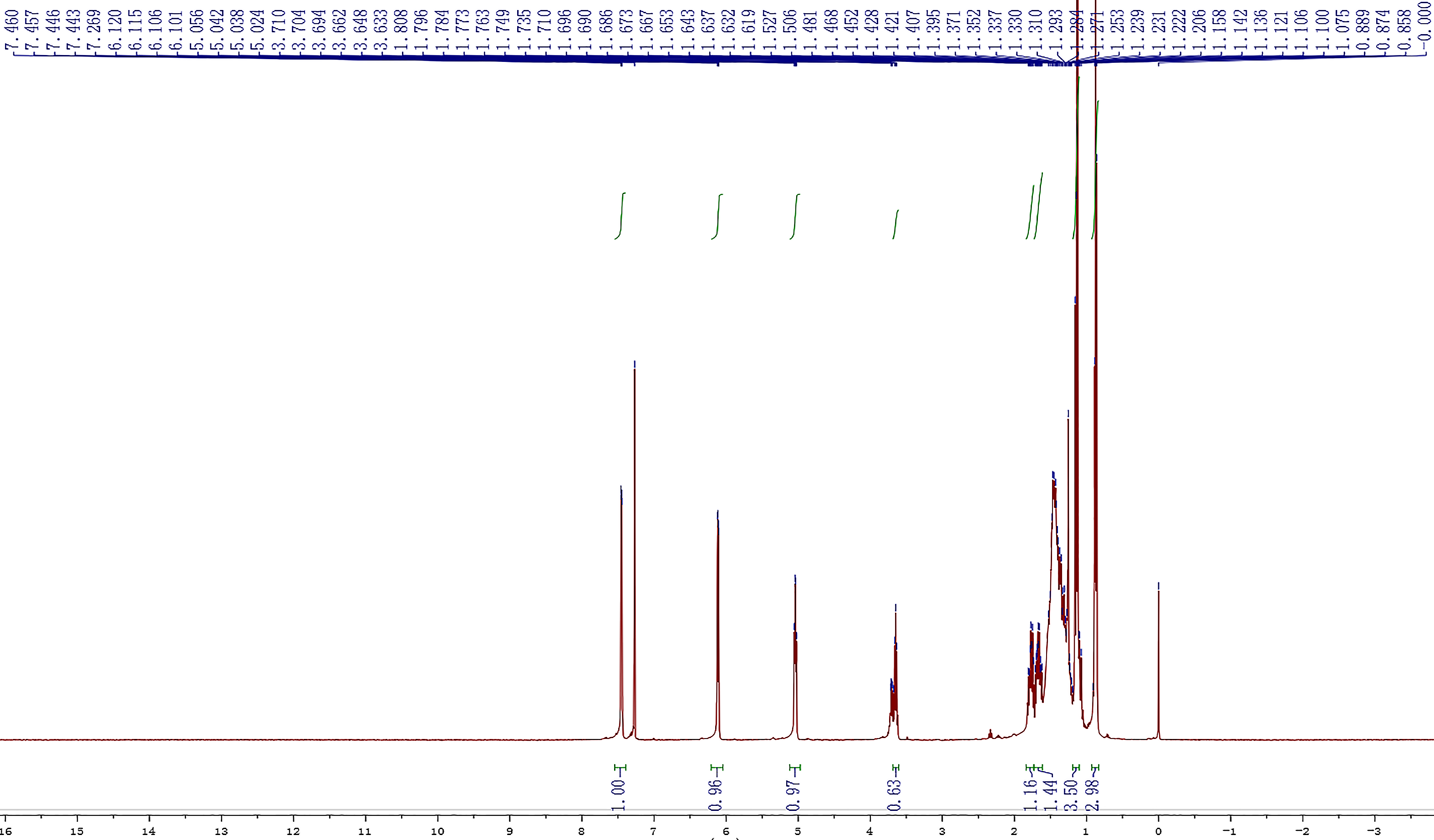

Supplement: Supplementary file 1 [file molecules-27-05649-s001.zip › S-PNG-8-15/Figure.S19 1H NMR (400 MHz) spectra of compound 4 in CDCl3.png]

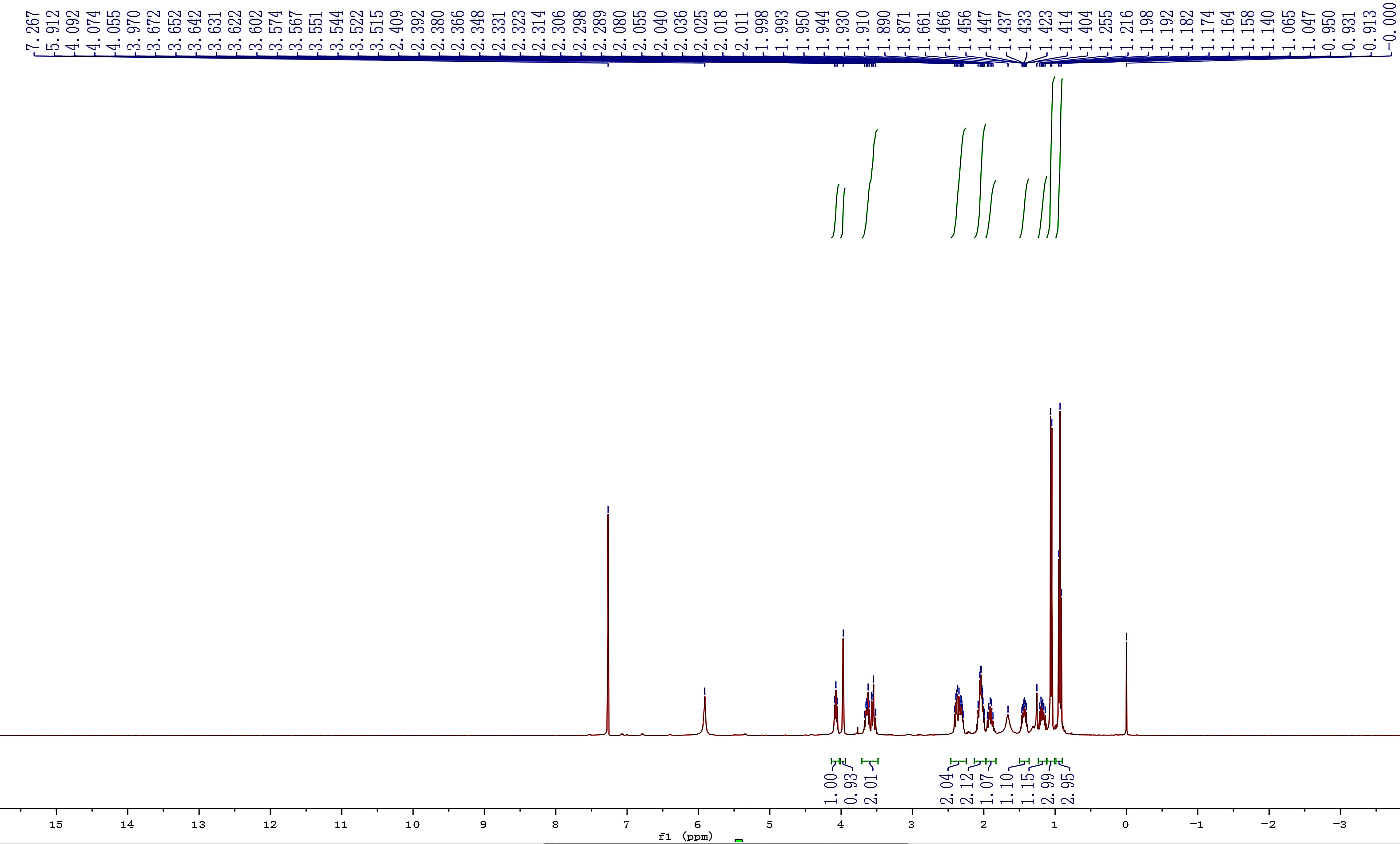

Supplement: Supplementary file 1 [file molecules-27-05649-s001.zip › S-PNG-8-15/Figure.S2 1H NMR (400 MHz) spectra of compound 1 in CDCl3.png]

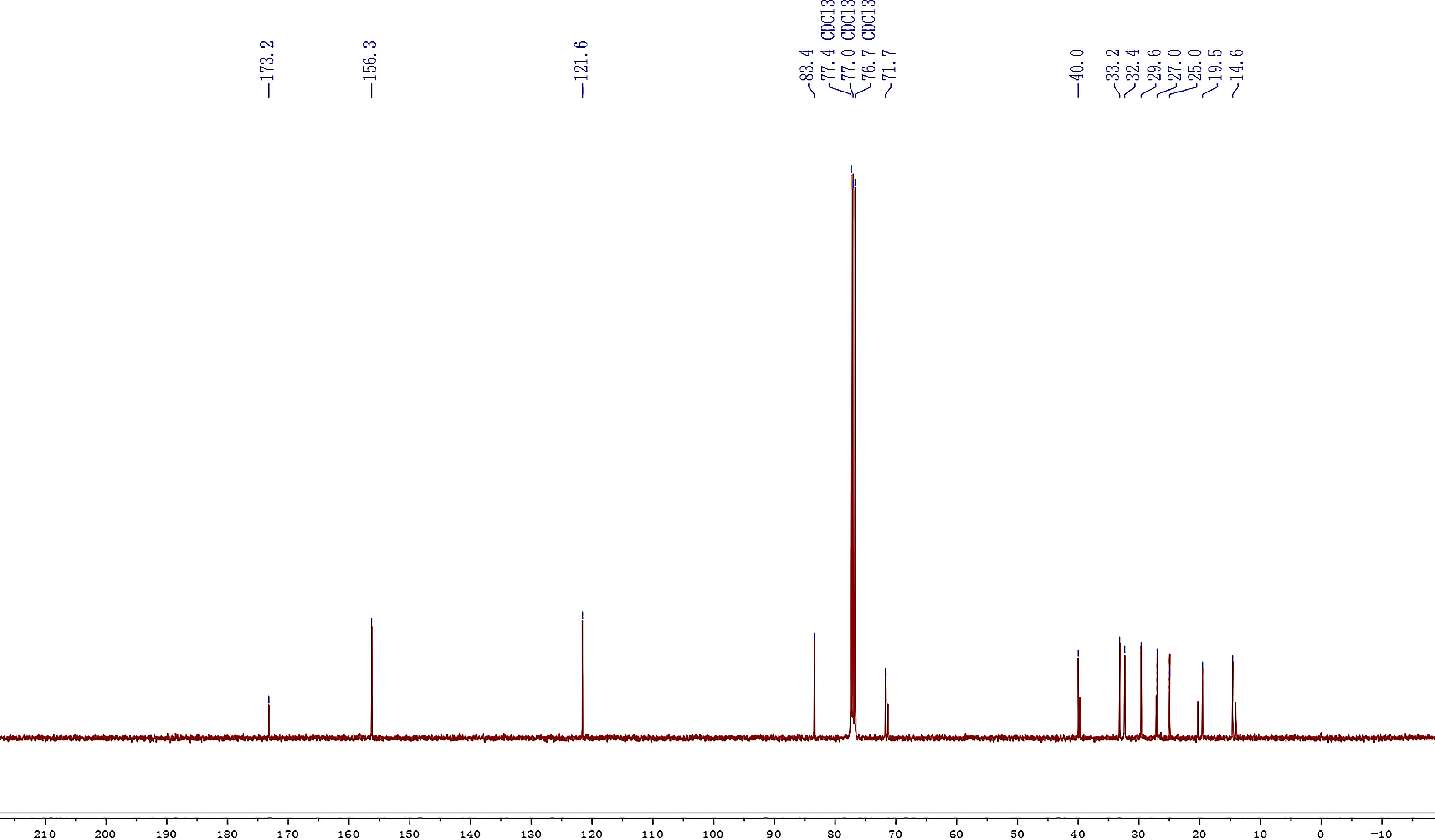

Supplement: Supplementary file 1 [file molecules-27-05649-s001.zip › S-PNG-8-15/Figure.S20 13C NMR (100 MHz) spectra of compound 4 in CDCl3.png]

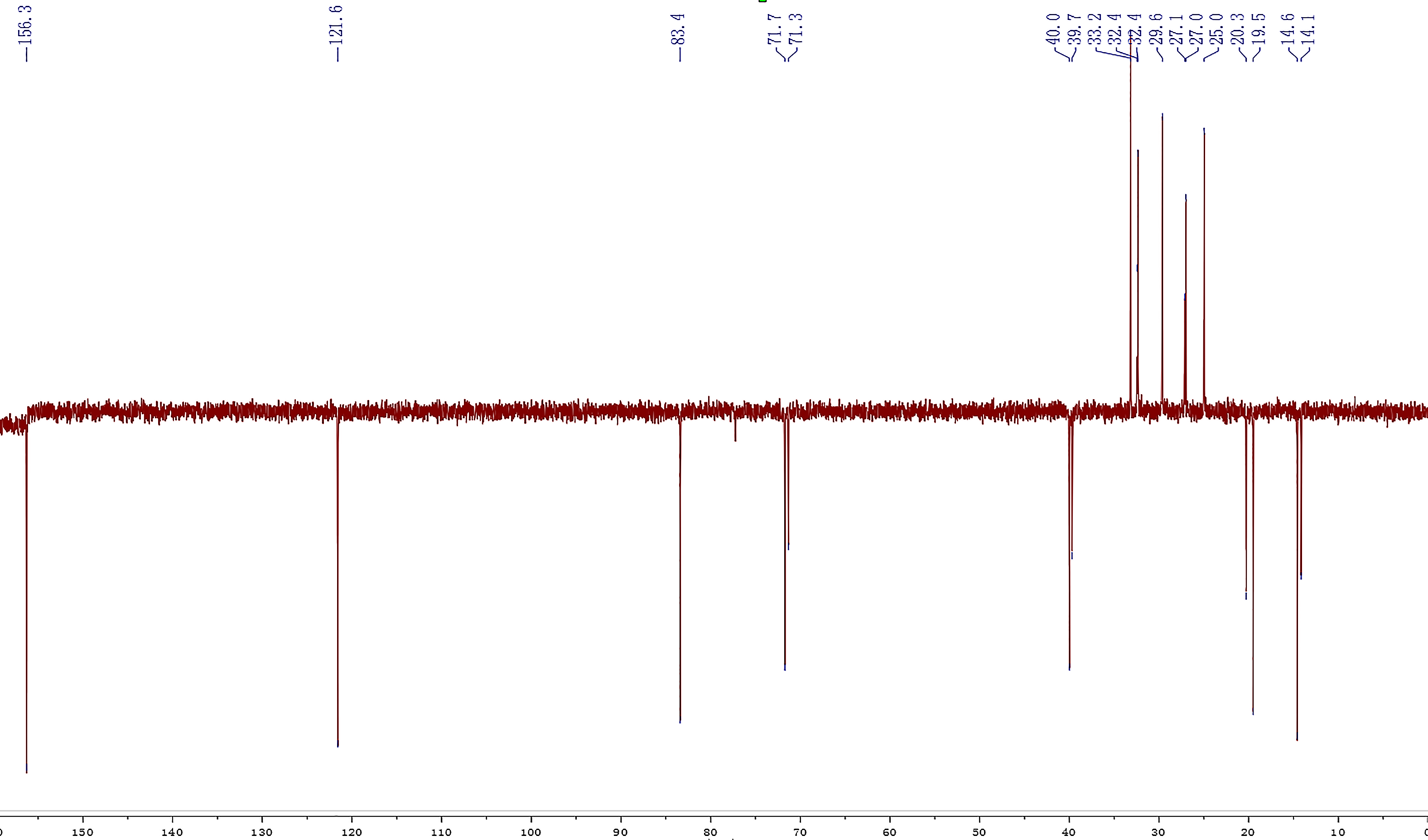

Supplement: Supplementary file 1 [file molecules-27-05649-s001.zip › S-PNG-8-15/Figure.S21 DEPT spectra of compound 4 in CDCl3.png]

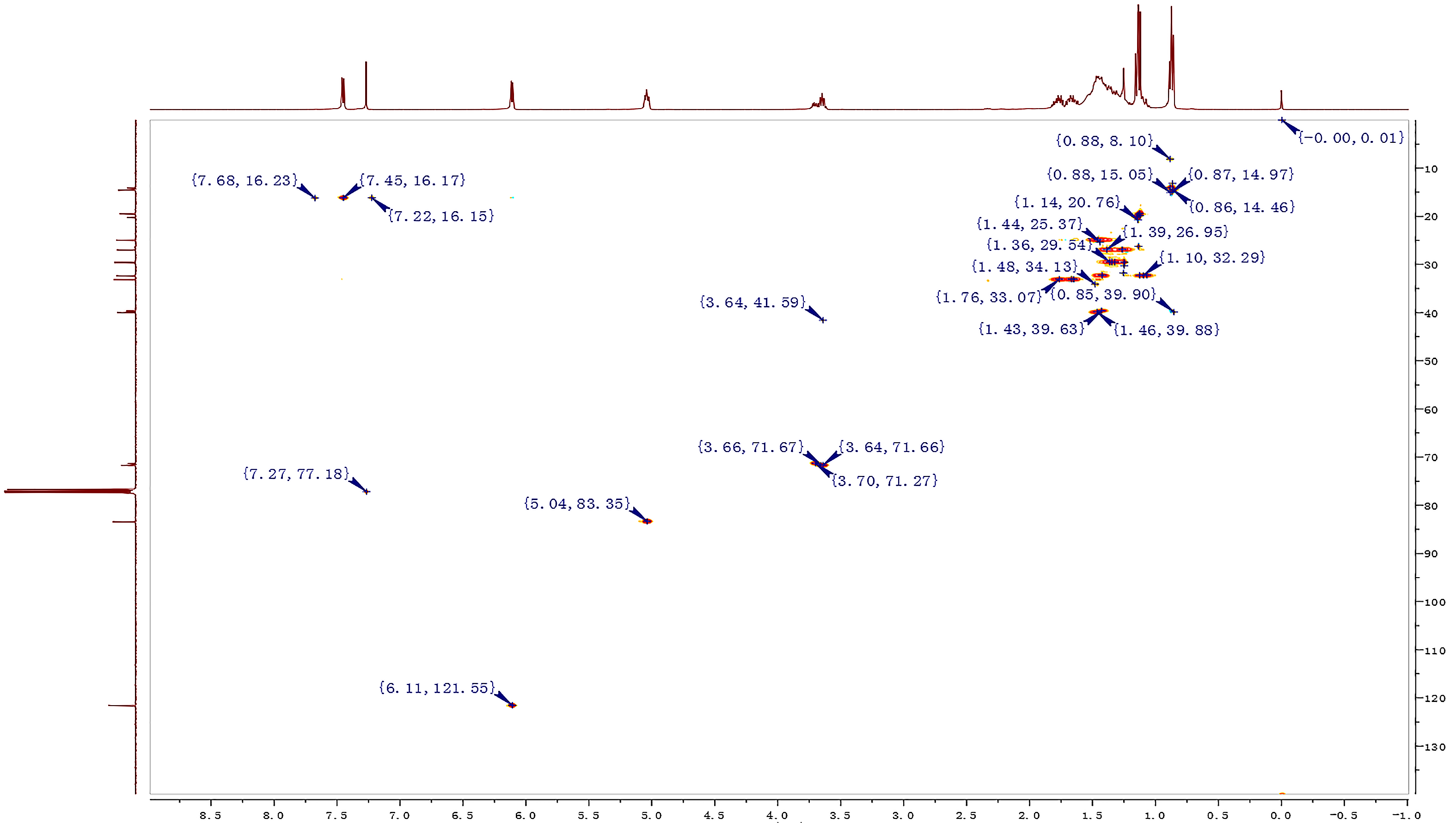

Supplement: Supplementary file 1 [file molecules-27-05649-s001.zip › S-PNG-8-15/Figure.S22 HSQC spectra of compound 4 in CDCl3.png]

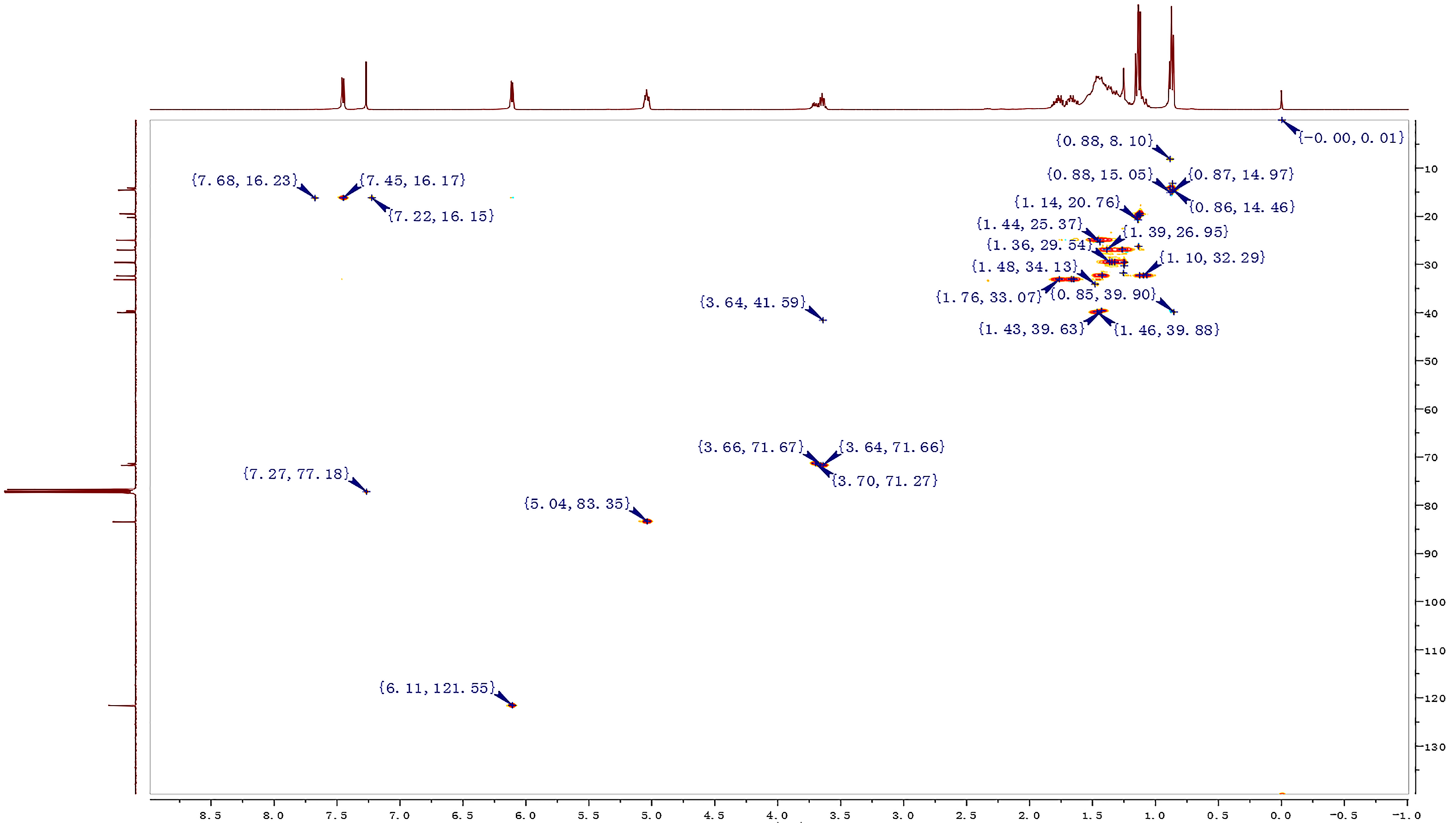

Supplement: Supplementary file 1 [file molecules-27-05649-s001.zip › S-PNG-8-15/Figure.S23 HMBC spectra of compound 4 in CDCl3.png]

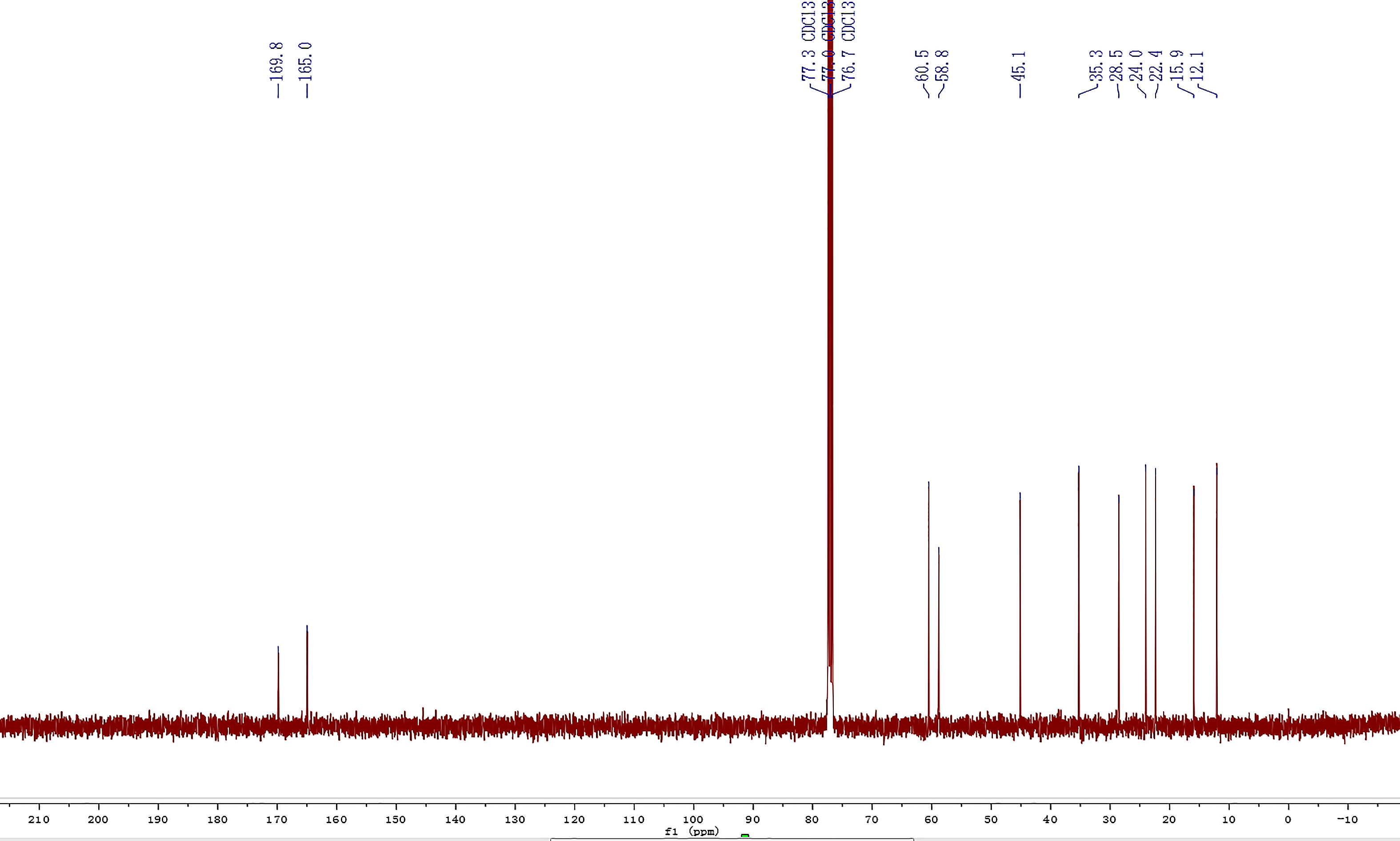

Supplement: Supplementary file 1 [file molecules-27-05649-s001.zip › S-PNG-8-15/Figure.S3 13C NMR (100 MHz) spectra of 1 in CDCl3.png]

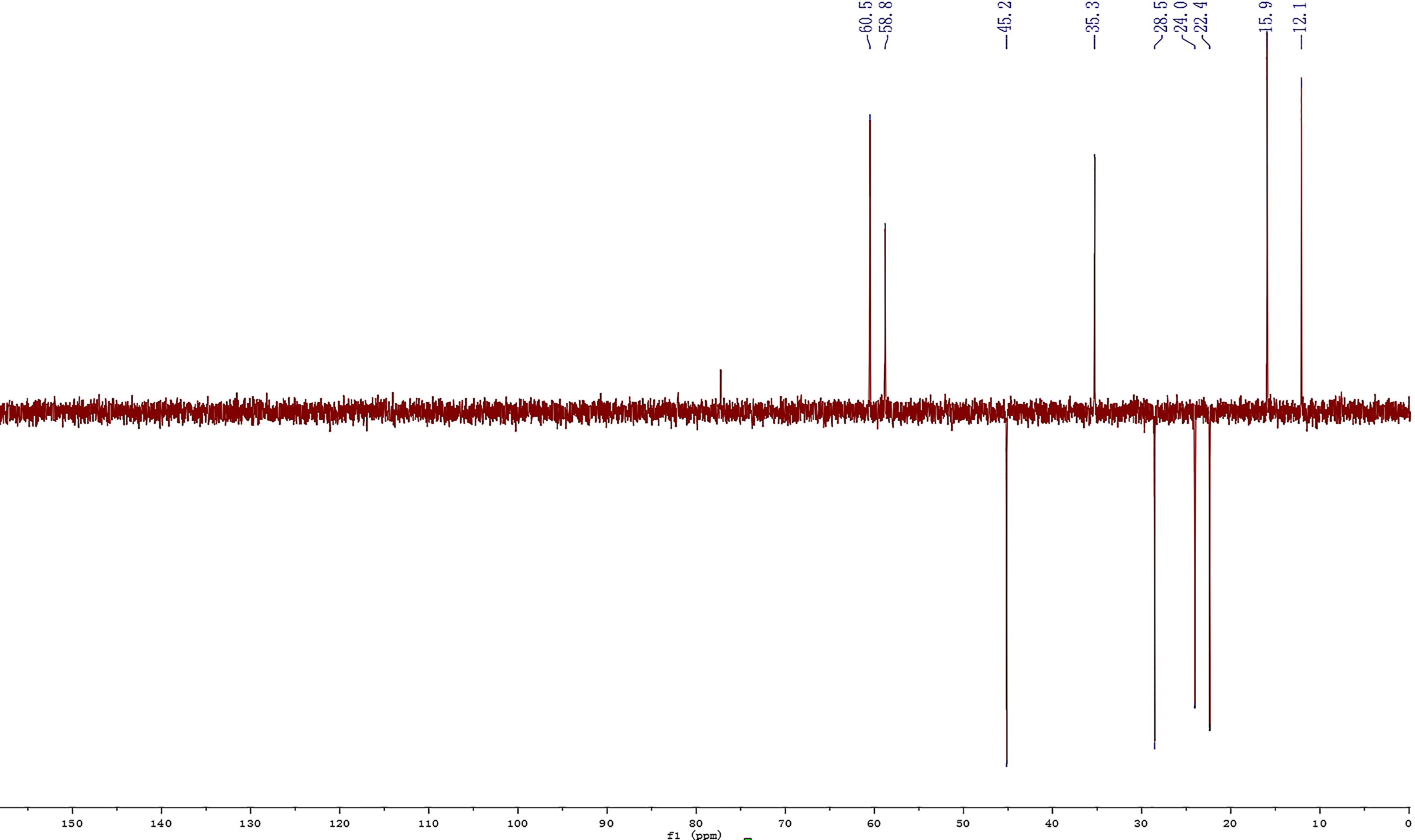

Supplement: Supplementary file 1 [file molecules-27-05649-s001.zip › S-PNG-8-15/Figure.S4 DEPT spectra of 1 in CDCl3.png]

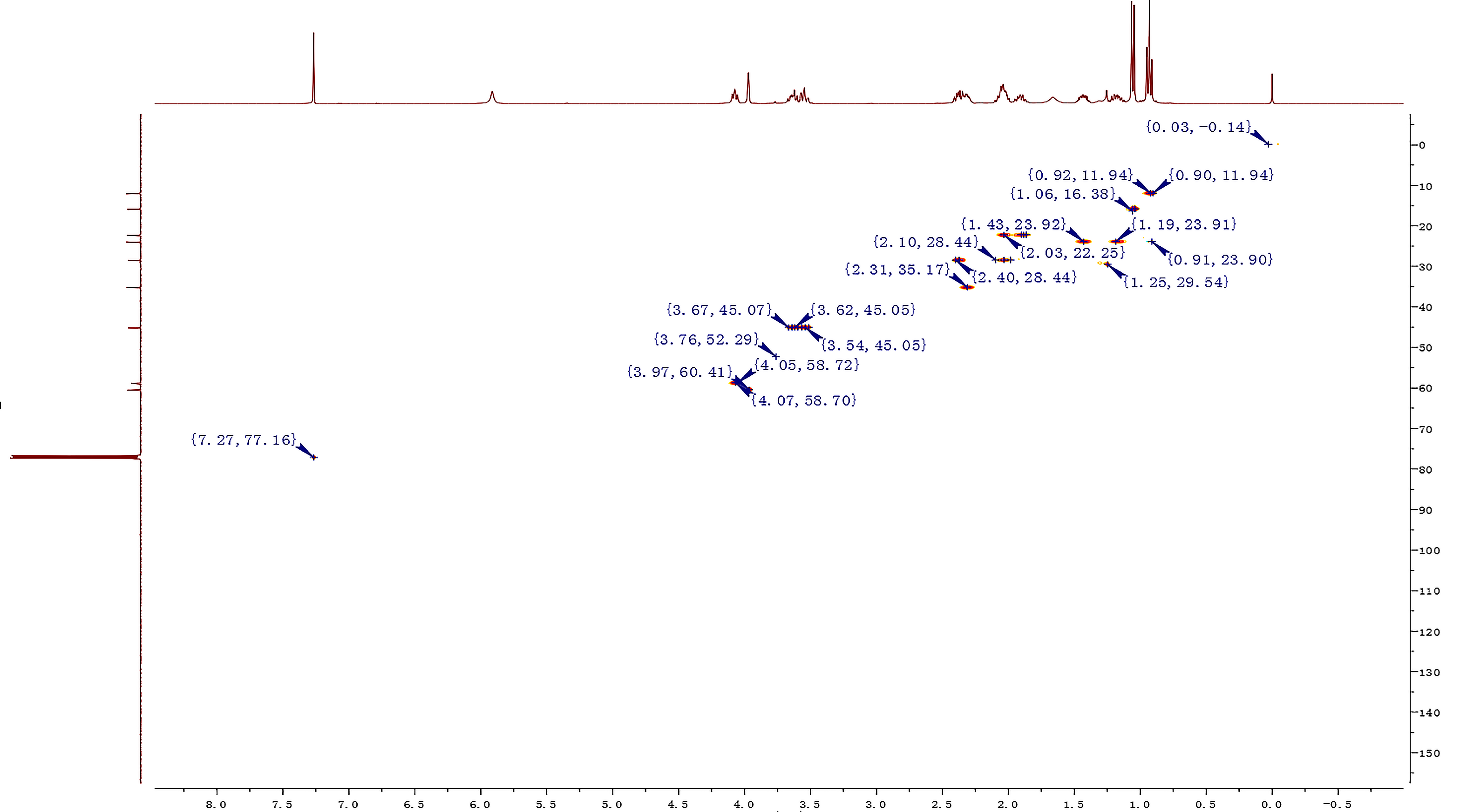

Supplement: Supplementary file 1 [file molecules-27-05649-s001.zip › S-PNG-8-15/Figure.S5 HSQC spectra of compound 1 in CDCl3.png]

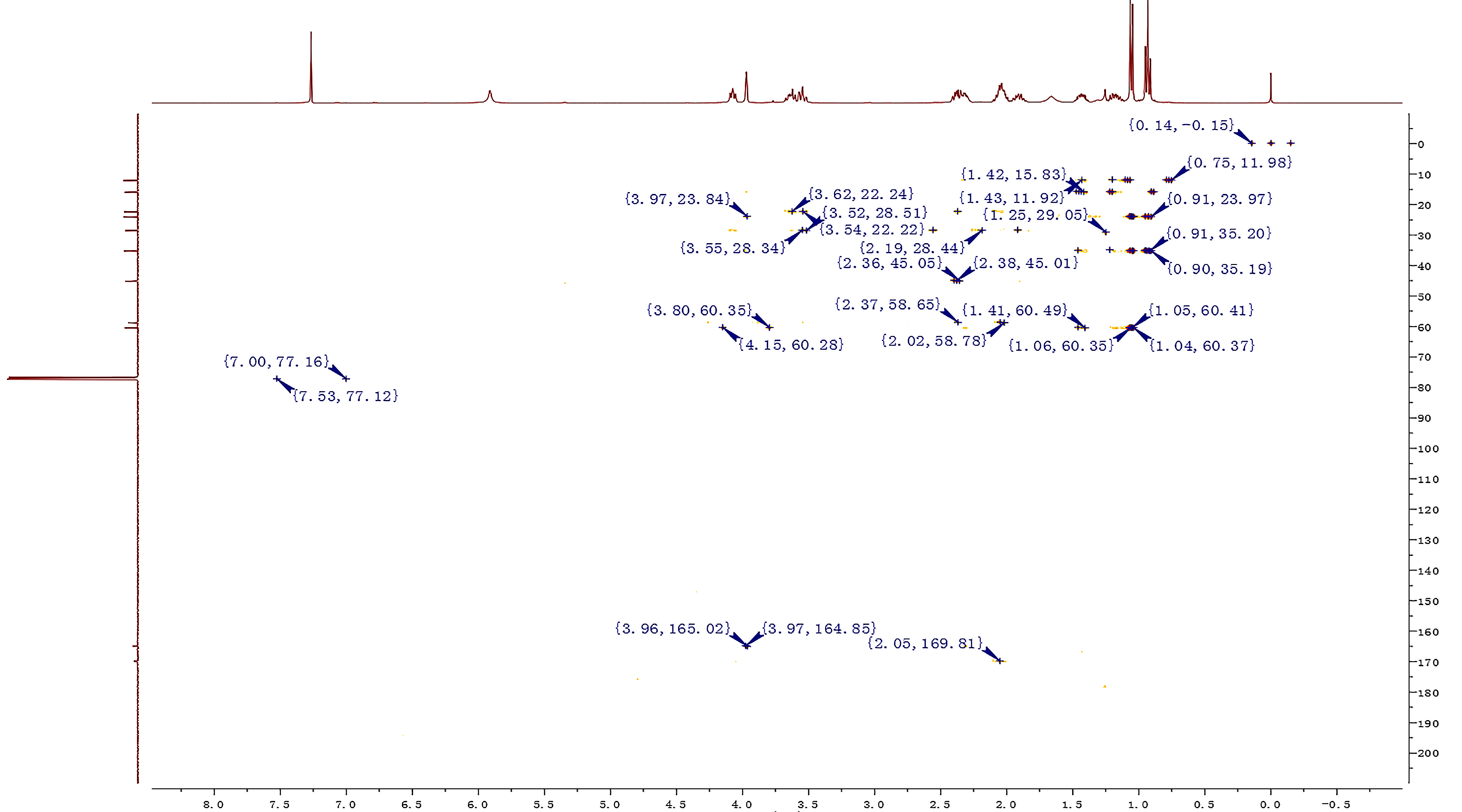

Supplement: Supplementary file 1 [file molecules-27-05649-s001.zip › S-PNG-8-15/Figure.S6 HMBC spectra of compound 1 in CDCl3.png]

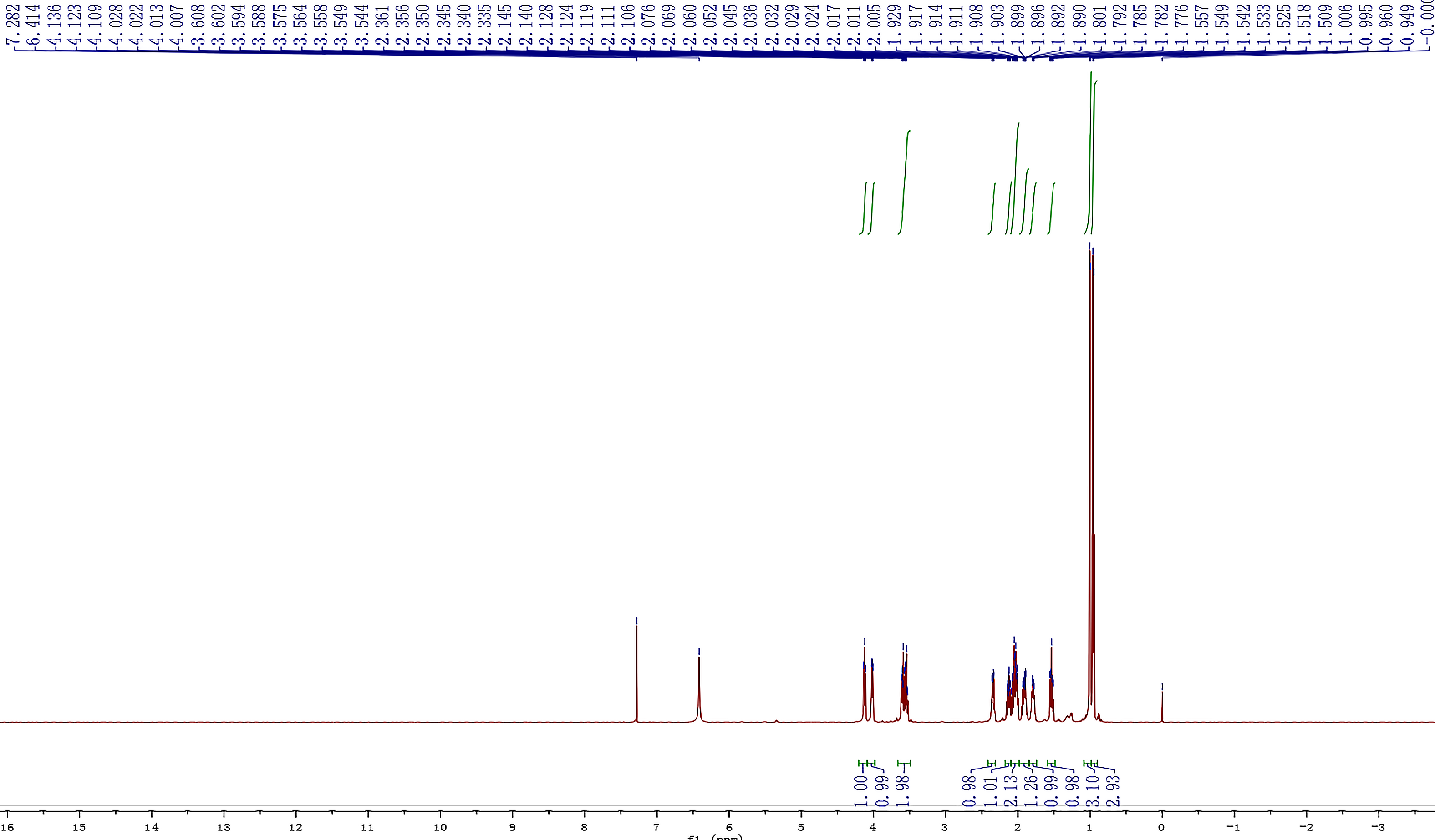

Supplement: Supplementary file 1 [file molecules-27-05649-s001.zip › S-PNG-8-15/Figure.S8 1H NMR (400 MHz) spectra of compound 2 in CDCl3.png]

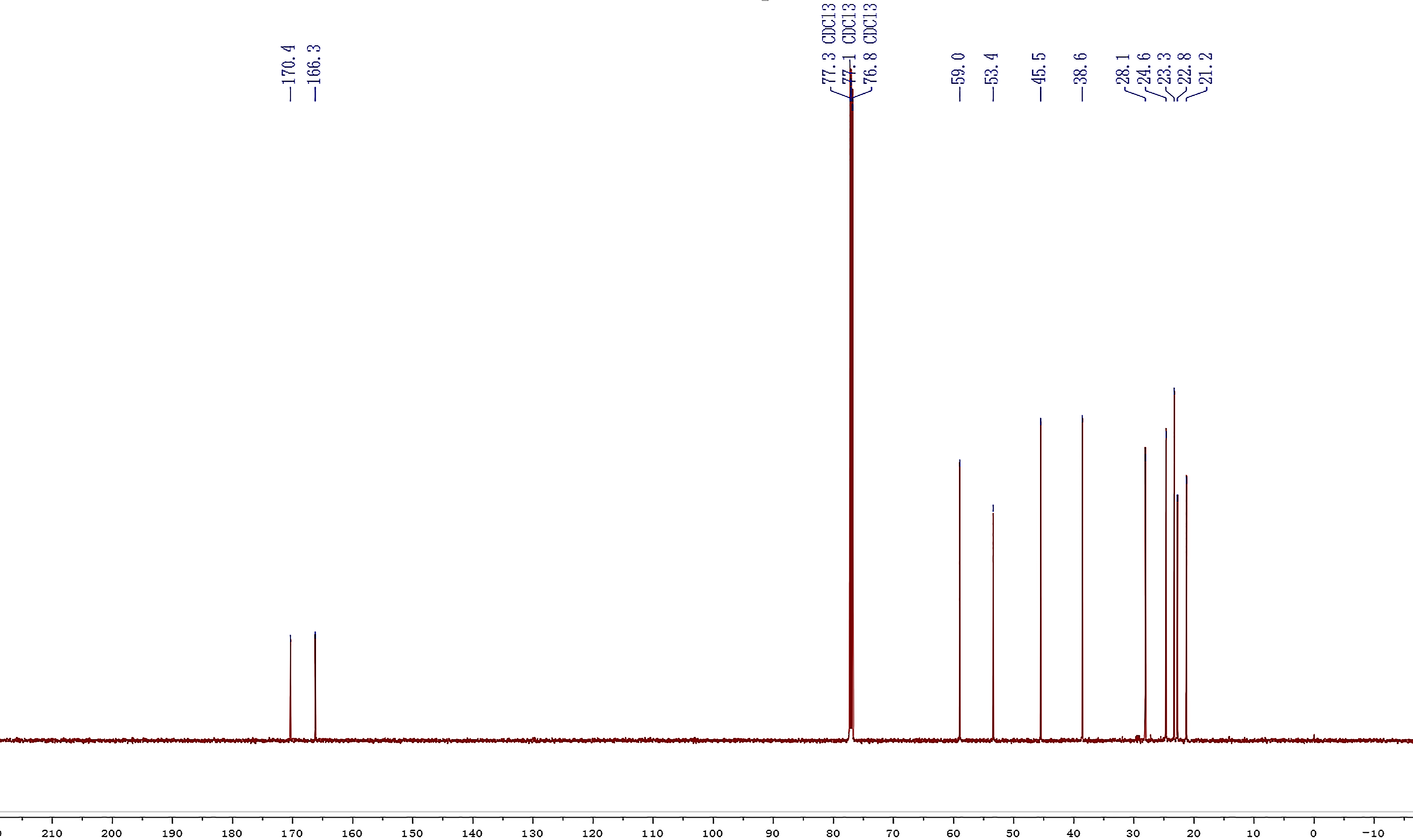

Supplement: Supplementary file 1 [file molecules-27-05649-s001.zip › S-PNG-8-15/Figure.S9 13C NMR (100 MHz) spectra of compound 2 in CDCl3.png]
